# Transcriptome Analysis of Salicylic Acid Treatment in *Rehmannia glutinosa* Hairy Roots Using RNA-seq Technique for Identification of Genes Involved in Acteoside Biosynthesis

**DOI:** 10.3389/fpls.2017.00787

**Published:** 2017-05-17

**Authors:** Fengqing Wang, Jingyu Zhi, Zhongyi Zhang, Lina Wang, Yanfei Suo, Caixia Xie, Mingjie Li, Bao Zhang, Jiafang Du, Li Gu, Hongzheng Sun

**Affiliations:** ^1^College of Agronomy, Henan Agricultural UniversityZhengzhou, China; ^2^College of Crop Sciences, Fujian Agriculture and Forestry UniversityFuzhou, China; ^3^School of Medicine, Henan University of Traditional Chinese MedicineZhengzhou, China

**Keywords:** *Rehmannia glutinosa*, acteoside, salicylic acid, RNA-seq, hairy root, biosynthesis

## Abstract

*Rehmannia glutinosa* is a common bulk medicinal material that has been widely used in China due to its active ingredients. Acteoside, one of the ingredients, has antioxidant, antinephritic, anti-inflammatory, hepatoprotective, immunomodulatory, and neuroprotective effects, is usually selected as a quality-control component for *R. glutinosa* herb in the Chinese Pharmacopeia. The acteoside biosynthesis pathway in *R. glutinosa* has not yet been clearly established. Herein, we describe the establishment of a genetic transformation system for *R. glutinosa* mediated by *Agrobacterium rhizogenes*. We screened the optimal elicitors that markedly increased acteoside accumulation in *R. glutinosa* hairy roots. We found that acteoside accumulation dramatically increased with the addition of salicylic acid (SA); the optimal SA dose was 25 μmol/L for hairy roots. RNA-seq was applied to analyze the transcriptomic changes in hairy roots treated with SA for 24 h in comparison with an untreated control. A total of 3,716, 4,018, and 2,715 differentially expressed transcripts (DETs) were identified in 0 h-vs.-12 h, 0 h-vs.-24 h, and 12 h-vs.-24 h libraries, respectively. KEGG pathway-based analysis revealed that 127 DETs were enriched in “phenylpropanoid biosynthesis.” Of 219 putative unigenes involved in acteoside biosynthesis, 54 were found to be up-regulated at at least one of the time points after SA treatment. Selected candidate genes were analyzed by quantitative real-time PCR (qRT-PCR) in hairy roots with SA, methyl jasmonate (MeJA), AgNO_3_ (Ag^+^), and putrescine (Put) treatment. All genes investigated were up-regulated by SA treatment, and most candidate genes were weakly increased by MeJA to some degree. Furthermore, transcription abundance of eight candidate genes in tuberous roots of the high-acteoside-content (HA) cultivar QH were higher than those of the low-acteoside-content (LA) cultivar Wen 85-5. These results will pave the way for understanding the molecular basis of acteoside biosynthesis in *R. glutinosa*, and can serve as a basis for future validation studies.

## Introduction

*Rehmannia glutinosa* is one of the most commonly used herbs in traditional Chinese medicine (TCM) and has been used in China for thousands of years. As a top-geoherb, Huai Dihuang (*Radix Rehmanniae* from Henan Province, China) is well-known domestically and abroad because of its favorable qualities (Huang et al., 2011). According to the Chinese medical classic “Shennong's Herba,” *R. glutinosa* is considered a “top grade” herb in China. Many clinical and experimental studies have reported that the root of *R. glutinosa* and its active components have pharmacological activities in the blood, cardiovascular system and endocrine, immune and nervous systems (Zhang et al., 2008). Previous phytochemical studies on the roots of *R. glutinosa* have led to the isolation and identification of saccharides, iridoids, sesquiterpenoids, phenylethanoid glycosides (PhGs), amino acids, inorganic ions, and other trace elements in this herb (Zhang et al., 2008; Li et al., 2015).

PhGs, including echinacoside, acteoside, and isoacteoside, are a class of polyphenolic compounds that are abundant in *R. glutinosa*. Acteoside, also called kusagin or verbascoside, is usually selected as a quality-control component in *R. glutinosa* herb and has been included in the Chinese Pharmacopeia since the 2010 edition. Pharmacological studies have shown that the bioactivities of acteoside include antioxidant, antinephritic, anti-inflammatory, hepatoprotective, immunomodulatory, and neuroprotective effects (He et al., 2011). Acteoside is widely distributed in dicotyledonous plants, such as Scrophulariaceae, Verbenaceae, and Oleaceae. Scarpati and Monache first isolated acteoside in 1963 from *Verbascum sinuatum* L. (Scarpati and Monache, 1963). To date, more than 150 plant species belonging to 20 families and 77 genera that contain acteoside have been reported (He et al., 2011). The level of acteoside varies greatly by plant family or genus, and even within a species. *Plantago lanceolata* had the highest acteoside concentration compared to that in five other species that were examined, whereas *Plantago schwarzenbergiana* had the lowest amount of acteoside (Janković et al., 2012). Interestingly, relative to the acteoside content in the leaves of *Stachys sieboldii*, the fleshy stem of *Cistanche salsa*, the fruit of *Forsythia suspense* and the flower of *Firmiana simplex*, the content of acteoside in *R. glutinosa* leaf was higher (Bian et al., 2010). However, acteoside generally accumulates to only low levels in plants, which limits its application in disease treatment.

Previous studies have focused on the molecular structure, content distribution, and biological effects of acteoside, but its biosynthesis pathway remains to be fully elucidated. Structurally, acteoside (C_29_H_36_O_15_) consists of two moieties, a hydroxytyrosol moiety and caffeoyl moiety, which are attached to a β-glucopyranose via a glycosidic bond (Jiménez and Riguera, 1994). The hydroxytyrosol moiety of acteoside is synthesized from tyrosine through dopamine, whereas the caffeoyl moiety of acteoside is synthesized from phenylalanine via a cinnamate pathway (Saimaru and Orihara, 2010; Alipieva et al., 2014). Experiments of isotope-labeled precursor feeding on *Olea europaea* cells have revealed that alternative biosynthesis pathways are available, e.g., from tyramine to acteoside via dopamine or from tyrosol to acteoside via hydroxytyrosol; however, the pathway from tyrosine to acteoside via DOPA and dopamine is the main biosynthesis pathway to acteoside (Saimaru and Orihara, 2010). Another study in *Syringa vulgaris* cell suspension cultures revealed that tyramine, tyrosol, and salidroside are more efficiently incorporated into acteoside than DOPA and dopamine (Ellis, 1983). However, the incorporation of salidroside into acteoside would be rare in *O. europaea* cell cultures (Saimaru and Orihara, 2010). This finding suggests that biosynthesis pathways for acteoside are not coincident among different plant species. Current knowledge of the biosynthesis pathway of acteoside is based primarily on feeding experiments; downstream intermediates are still unknown without further research.

Currently, comparative transcriptomics is a method of choice to unravel the biosynthesis pathways of secondary metabolites on a transcriptome-wide scale in non-model plant species (particularly medicinal plants), for which the complete genome sequences and annotation are not yet available. *De novo* transcriptome sequencing and characterization has been performed successfully for *Taxus mairei* (Hao et al., 2011), *Scutellaria baicalensis* (Yuan et al., 2012a), *Glycyrrhiza uralensis* (Ramilowski et al., 2013), *Lycium chinense* (Zhao et al., 2013), *Litsea cubeba* (Han et al., 2013), *Opium Poppy* (Gurkok et al., 2014), *Panax notoginseng* (Liu et al., 2015), and *Gastrodia elata* (Tsai et al., 2016).

We successfully established hairy root cultures of *R. glutinosa*, which provided a new approach to obtain and screen key genes in the acteoside biosynthesis pathway. Little information exists on the relationship between the yield of acteoside and expression levels of key genes encoding enzymes involved in acteoside biosynthesis when they are induced by elicitors. In this study, the effects of chemical elicitors, including salicylic acid (SA), methyl jasmonate (MeJA), silver nitrate (Ag^+^), and putrescine (Put), on the production of acteoside in a *R. glutinosa* hairy root culture line were studied. We performed transcriptome analysis using RNA-seq to analyze the transcriptome of *R. glutinosa* hairy roots, focusing on the molecular basis of acteoside biosynthesis under SA treatment. Simultaneously, expression levels of candidate genes involved in the biosynthesis of this component were investigated.

## Materials and methods

### Plant materials

For aseptic seedling culture, tuberous roots of *R. glutinosa* Libosch “Wen 85-5” were surface sterilized with 0.1% mercuric chloride and cultured on hormone-free MS agar medium (Murashige and Skoog, 1962). The MS propagation medium contained 30 g/L sucrose and 9 g/L agar. The seedlings were maintained at 26°C under a 14 h light/10 h dark photoperiod in a growth chamber. Tuberous roots of two *R. glutinosa* cultivars Wen 85-5 and QH were planted on April 20, 2015. The fields were maintained with locally standard production conditions. The tuberous roots (TR), stems (S), tender leaves (TL), unfolded leaves (UL), and senescing leaves (SL) of the Wen 85-5 plants were collected from six plants grown in the field for 4 months. The TR of QH plants were collected at the same time. All the tissues from the six plants were separately pooled for each determination. The experiments were repeated in triplicate.

### Establishment of hairy root cultures

The culture of *Agrobacterium rhizogenes* strain ACCC10060 was initiated from glycerol stock and grown overnight at 28°C with shaking (180 rpm) in liquid YEB medium to the mid-log phase (*OD*_600_ = 0.8). Excised leaves of Wen 85-5 were dipped into *A. rhizogenes* ACCC10060 liquid inoculation medium for 8 min to infect leaf explants of 25-day-old seedlings, blotted dry on sterile filter paper, and incubated in the dark at 26°C on solid MS agar medium containing 100 μmol/L acetosyringone (AS). After co-cultivation for 2 days, the leaf explants were transferred to liquid MS agar medium containing 500 mg/L cefotaxime (Cef) and 100 μmol/L AS for hairy root induction. Numerous hairy roots were observed emerging from the wound sites after ~2–4 weeks. When the hairy roots had grown to a length of 2–3 cm, they were separated from the explants and cultured in dark at 26°C on MS agar medium with a gradual decrease of Cef at a 10 day interval to obtain bacteria-free culture. The hairy root segments were checked for bacterial contamination by culturing them in MS medium without Cef. The rapidly growing hairy root culture line was transferred to 50 mL of MS liquid medium in 100 mL flasks. The hairy root cultures were maintained at 26°C on a gyratory shaker at 120 rpm in the dark.

For confirmation, isolation of total DNA from bacterium-free *R. glutinosa* hairy roots and non-transformed (NT) roots was conducted using a modified CTAB method (Cuc et al., 2008). Polymerase chain reactions (PCR) were performed for *rolB* and *rolC* using specific primers recognizing *rolB* (5′-GCTCTTGCAGTGCTAGATTT-3′; 5′-GAAGGTGCAAGCTACCTCTC-3′) and *rolC* (5′-CTCCTGACATCAAACTCGTC-3′; 5′-TGCTTCGAGTTATGGGTACA-3′). The amplification system and procedure were the same as in Tong et al. (2012), except for the primer annealing temperature, which was 58°C.

### Preparation and addition of four elicitors

A solution of 10 mmol/L SA (Sigma-Aldrich) was prepared by dissolving 0.0414 g SA powder in 30 mL sterile distilled H_2_O. MeJA (Sigma, China) was dissolved in DMSO to a concentration of 100 mmol/L. AgNO_3_ (Ag^+^, 0.3397 g) was dissolved in 20 mL distilled H_2_O with a concentration of 100 mmol/L. Put (Sigma, China) was dissolved in sterile distilled H_2_O to a concentration of 10 mmol/L. All the solutions were filter sterilized through 0.22 μm filters and added to cultures to the desired final concentrations. Experiments were conducted in a complete random block design with three replicates of each treatment and three concentration levels (high, medium, and low concentration) for each elicitor, generating a total of 39 experimental units (10 treatments, 13 × 3 replicates). The final concentrations were 10, 25, and 40 μmol/L for SA and 5, 15, and 25 μmol/L for MeJA, Ag^+^, and Put, respectively. These four stock solutions of elicitors were stored at 4°C prior to use. For elicitor treatments, 0.1 g (fresh weight) hairy roots were incubated in 50 ml liquid MS medium in 100 ml Erlenmeyer flasks. Sterilized solutions were individually added to 20-day-old hairy root culture media at the designed concentrations to investigate their effects on hairy root growth and acteoside content. Water was added as a negative control. After 10 days of treatment with different elicitors, hairy roots were harvested for determination of dry weight and acteoside content. After 0, 12, and 24 h treatment with SA, hairy roots were collected for determination of acteoside content and RNA-seq analysis. The 0, 3, 9, 12, and 24 h SA-treated hairy roots were used for expression analysis. All treatments were performed in triplicate, and the results were averaged.

### RNA extraction, library preparation, and illumina sequencing

For RNA-seq analysis, pooled hairy roots from three replicate cultures were used to prepare an RNA sample for each treatment. Frozen hairy root tissue (100 mg) was ground to a powder in liquid nitrogen. Total RNA was isolated with TRIzol® Reagent (Invitrogen) as described by the manufacturer's protocol, then treated with RNase-free DNaseI (Invitrogen). The RNA quality and quantity were determined with a Nanodrop™ 2000 spectrophotometer (Thermo Fisher Scientific, USA) and a Bioanalyzer 2100 (Agilent, USA). Library preparation and Illumina sequencing were performed using an Illumina HiSeq™ 2000 from BGI-Tech (Shenzhen, China; Project ID: F15FTSCCKF2309). Transcripts from tuberous roots and leaves transcriptomes of *R. glutinosa* (Project ID: F13FTSCCKF1467) were used as references for read mapping and gene annotation.

### RNA-seq data analysis

The raw reads were subjected to quality control (QC) analysis to identify high-quality sequencing data and clean reads. Filtered clean reads from each sample were then separately aligned to the reference transcripts of *R. glutinosa* using the Bowtie2 software (Langmead et al., 2009) and used to estimate the abundance of gene transcripts using the RSEM method (Li and Dewey, 2011), measured as fragments per kilobase of transcript per million fragments sequenced (FPKM) (Trapnell et al., 2010). Differentially expressed genes were identified based on the method described by Audic and Claverie (1997). We used a false discovery rate (FDR) of ≤0.001 and an absolute value of FPKM fold-change of ≥2 as the thresholds to evaluate the significance of differentially expressed transcripts (DETs). The expression profiles of DETs from different samples were analyzed by hierarchical clustering, and a heat map of expression values was generated using the T-MeV 4.9.0 software (Howe et al., 2011). Annotation analyses of DETs were performed using WEGO software (Ye et al., 2006) for GO term functional classification, and pathway enrichment analysis of DETs was performed based on the Kyoto Encyclopedia of Genes and Genomes (KEGG) database (Kanehisa et al., 2008).

### Quantitative real-time RT-PCR assay

Total RNA was extracted using TRIzol reagent (Invitrogen) according to the manufacturer's protocol. Total RNA samples were treated extensively with RNase-free DNase I (Invitrogen) to remove any contaminating genomic DNA. cDNA was obtained from 1 μg of total RNA using a PrimeScript™ II 1st Strand cDNA Synthesis Kit (TaKaRa Bio, Dalian). Quantitative real-time PCR (qRT-PCR) assays were performed using SYBR® Premix Ex Taq™ II (Tli RNaseH Plus) (Takara Bio, Dalian) on a Bio-Rad iQ5 Real-Time PCR System (Bio-Rad, USA) as described by Wang et al. (2015). The *RgTIP41* gene (GenBank accession number KT306007) was used as the reference gene to calculate relative expression levels based on the 2^−ΔΔCt^ method (Schmittgen and Livak, 2008). Data from three biological replicates were analyzed using analysis of variance (ANOVA) followed by Student's *t*-test (*p* < 0.05). The primers used are listed in Table S1.

### HPLC analysis

Dried and powdered *R. glutinosa* hairy roots (0.8 g) were extracted with methanol for 1.5 h at room temperature, and the weight loss was made up with methanol. After filtration, the 20 mL extract was vacuum evaporated to dryness and the residue was dissolved in 5 mL acetonitrile-acetic acid solution (16:84, v/v), and then was determined by HPLC. Targeted analysis of acteoside was performed on an Agilent 1200 HPLC with a C18 column (4.6 × 250 mm, 5 μm) at 30°C. The mobile phase was acetonitrile-acetic acid solution (16:84, v/v) and was run at 1 mL/min. The injection volume was 20 μL for each sample, and detector wavelength was 334 nm. The reference standard of acteoside was purchased from Chengdu Must Bio-Technology Co., Ltd (Sichuan, China). Acteoside concentration was calculated by interpolating the peak area with a calibration curve obtained from standard purified compounds in the range of 0.038–0.56 mg/mL and expressed as mg/g of hairy root dry weight or mg/L of liquid culture.

## Results

### Elicitor effects on growth and acteoside accumulation in *R. glutinosa* hairy roots

**Figure 1:**
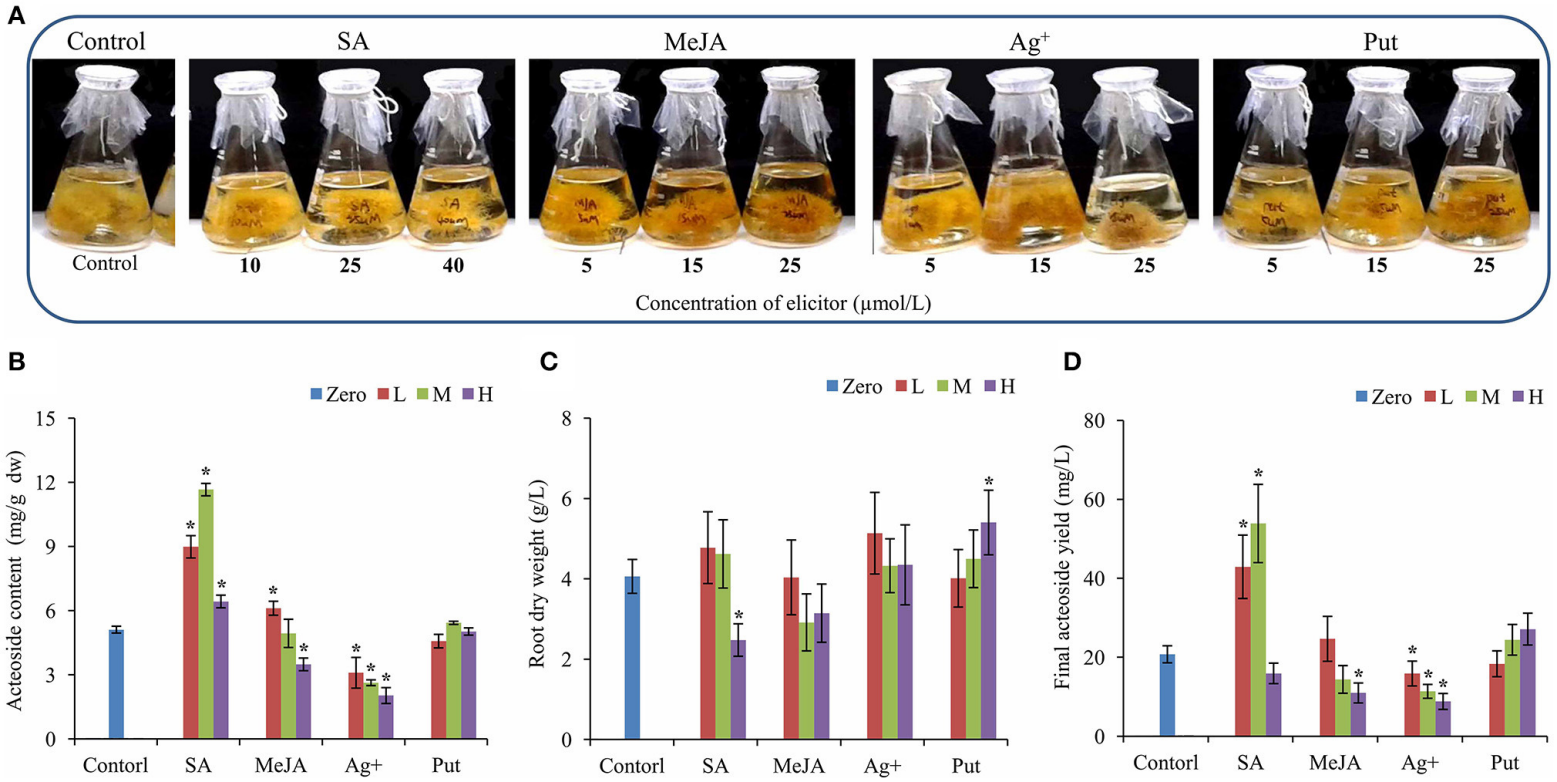
**Effects of elicitors on root growth and acteoside content of ***R. glutinosa*** hairy roots. (A)** Growth status of hairy roots with elicitors. **(B–D)** Root dry weight **(A)**, Acteoside content **(B)** and final acteoside yield **(C)** of the *R. glutinosa* hairy roots after SA, Ag^+^, MeJA, and Put treatment. The vertical bars show the *SD* values (*n* = 3). The asterisks indicate statistically significant differences at *p* < 0.05. L, lower concentration; M, middle concentration; H, higher concentration.

The hairy root lines of *R. glutinosa* were established by *Agrobacterium rhizogenes* infection, and its state was confirmed by PCR for the amplification of two segments of (423 and 626 bp) corresponding to the bacterial *rolB* and *rolC* genes, respectively (Figure S1). After 30 days of growth in liquid MS medium, the biomass of hairy roots had essentially reached a maximum (Figure S2), and the roots were used for acteoside determination. To investigate the effects of abiotic elicitors on acteoside accumulation, 20-day-old *R. glutinosa* transgenic hairy roots were treated with SA, MeJA, silver nitrate (Ag^+^) and Put and then cultivated continuously for 10 days (Figure 1A). Relative to control untreated hairy roots, SA or MeJA both induced a significant increase in acteoside content at an appropriate concentration (Figure 1B). However, SA appeared to be more effective in triggering acteoside accumulation than MeJA. An approximate concentration of 25 μmol/L SA was the most efficient and acteoside content under this treatment (11.66 ± 0.19 mg/g dw) was 2.28-fold that of the control. Because the final biomass of hairy roots after SA elicitation was substantially the same and did not differ from that of control hairy roots (Figure 1C), the total amount of acteoside extracted from 1 L of SA hairy root liquid culture (53.87 ± 9.91 mg/L) was significantly higher than the amount in untreated hairy roots (20.75 ± 2.15 mg/L) (Figure 1D). However, Ag^+^ inhibited acteoside accumulation, and the content of acteoside was significantly lower with increasing concentrations of Ag^+^ (Figure 1B). When 25 μmol/L Put was added, the biomass was increased by 33% relative to the control, while no significant increase of content and a final yield of acteoside was abserved (Figures 1C,D). It can be concluded that the best elicitor for further enhancement of acteoside accumulation in transgenic *R. glutinosa* hairy root line Wen 85-5 is SA at a concentration of 25 μmol/L.

Elicitors can trigger physiological responses and secondary metabolites accumulation in a short time (Sá et al., 1992). To study the short-term effect of SA treatment on acteoside accumulation, hairy roots were collected at 0, 12, and 24 h after SA treated. The result showed that acteoside accumulation were significantly improved in 12 and 24 h SA treated hairy roots compared to untreated hairy roots (Figure S3).

### Overview of differentially expressed genes in *R. glutinosa* hairy roots after SA treatment

As the genome sequence of *R. glutinosa* is not yet available and we were most interested in transcribed genes, we conducted transcriptomic analysis of the induced *R. glutinosa* hairy root cultures. To investigate the transcriptome response to SA induction in the hairy roots of *R. glutinosa*, the Illumina HiSeq 2000 platform was used to perform high throughput tag-seq analysis on *R. glutinosa* hairy root libraries constructed at 3 time points before and during the 24 h SA treatment period. The samples were collected at 0, 12, and 24 h after SA treatment. The major characteristics of these libraries are summarized in Table 1. Approximately 13 million sequence tags per library were obtained. Prior to mapping these tag sequences to the reference transcriptome sequences, adaptor, low quality and single copy tags were removed, resulting in a total of more than 12.7 million clean sequence tags per library. The RNA-seq reads data of three samples have been deposited in NCBI's Sequence Read Archive (SRA) under accession number SRP103641, including SRR5438036, SRR5438037, and SRR5438042.

**Table 1:** **Alignment of Illumina HiSeq 2000 reads onto the ***R. glutinosa*** reference transcriptome**.

**Sample**	**0 h**	**12 h**	**24 h**
Total raw reads	13042810	13042437	13043134
Total clean raw reads	12770061 (97.9%)	12711339 (97.46%)	12778530 (97.97%)
Total mapped clean reads	10419908 (81.6%)	10512337 (82.7%)	10878004 (85.13%)
Perfect match	6289451 (49.25%)	6551141(51.54%)	6894340 (53.95%)
Unique match	6084778 (47.65%)	6030483 (47.44%)	6339325 (49.61%)
Multi-position match	4335130 (33.95%)	4481854 (35.26%)	4538679 (35.52%)
Mapped reference genes	48246 (73.47%)	48378 (73.68%)	49009 (74.64%)
Unigenes with FPKM ≥ 2.0	33765 (69.98%)	34138 (70.56%)	35104 (71.63%)

Using our assembled *R. glutinosa* unigenes as the reference transcriptome, 47.44–49.61% of the unique clean reads (6.03–6.34 million) were uniquely mapped, representing 57.37–58.39% of the total mapped reads. In the corresponding individual samples, 48,246, 48,377, and 49,009 reference unigenes were identified, indicating that our sequencing depth was sufficient to approach saturation.

**Figure 2:**
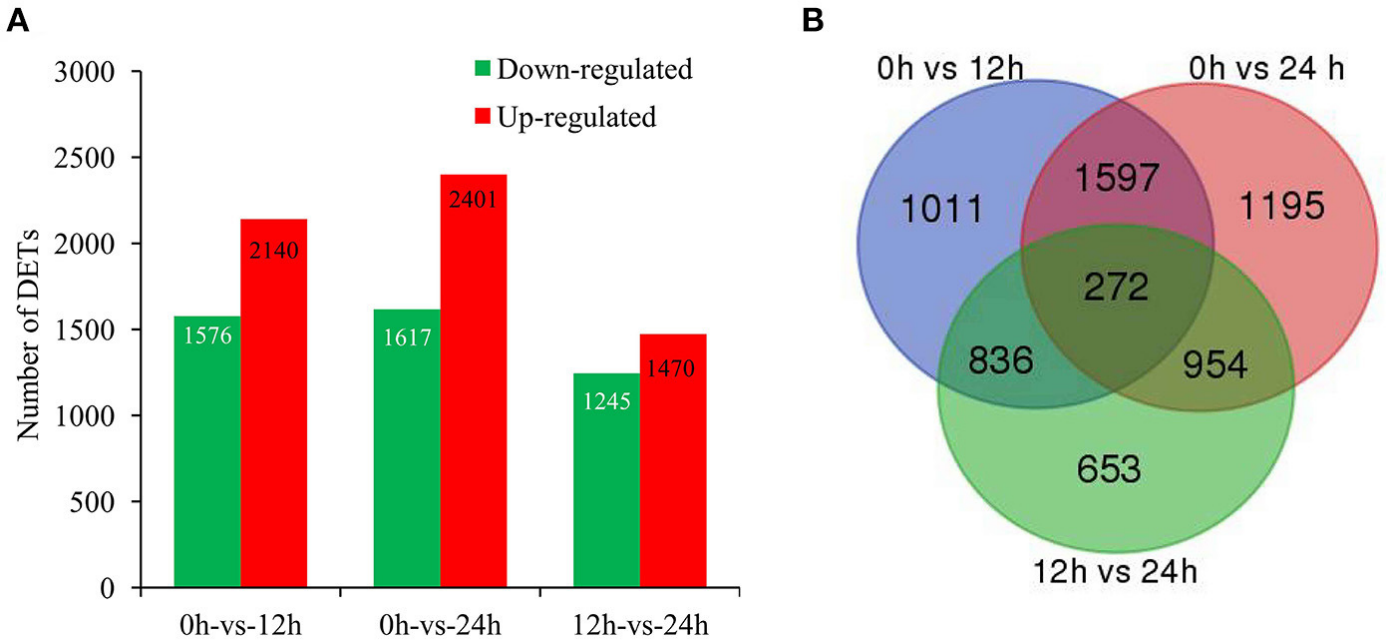
**Genes significantly differentially expressed in response to SA treatment. (A)**, The up- and down-regulated transcript numbers during SA treatment. *X*-axis represents pairwise and *Y*-axis means number of screened DETs. Green bar denotes down-regulated genes and red bar for the up-regulated. **(B)**, The Venn diagram analysis of the quantity of the DETs identified at different time points of SA treatment.

To identify the genes with significant change in expression level during SA treatment, DETs between 0 h and the other two libraries (12 and 24 h) were identified. Our digital expression analysis identified 2,715–4,018 DETs with at least 2-fold difference in expression levels and a FDR < 0.001 during 24 h SA treatment (Figure 2A). The differential expression patterns among libraries revealed that the largest differences occurred between 0 and 24 h, and 2,401 up-regulated and 1,617 down-regulated DETs were identified at 24 h; whereas the smallest differences existed between 12 and 24 h, for which only 1,470 up-regulated and 1,245 down-regulated DETs were identified (Figure 2A). Furthermore, we could identify 272 common genes through a Venn diagram of the three comparison periods in SA treatment (Figure 2B). In detail, 90 common up-regulated genes were found among the three comparisons, including alcohol dehydrogenase gene (*ALDH*, CL6693.Contig36), 4-coumarate-CoA ligase gene (*4CL*, CL929.Contig4), and cinnamoyl-CoA reductase gene (*CCR*, Unigene7) (Figure S4A; Table S2), etc. In contrast, only 54 common down-regulated genes were found in the same comparisons (Figure S4B; Table S2).

To further validate the results of mRNA sequencing, 10 transcripts were randomly selected along with their specific primers for qRT-PCR analysis (Table S1). All 10 transcripts showed similar expression patterns to the *in silico* differential analysis results from DET sequencing. Expression levels measured by qRT-PCR were strongly correlated with those of DETs identified by mRNA-seq (*r* = 0.8182; Figure S5). In addition, the production of expected fragment sizes using designed primers also supported the reliability of the *de novo* assembly.

### Functional analysis of differentially expressed genes based on RNA-seq data

**Figure 3:**
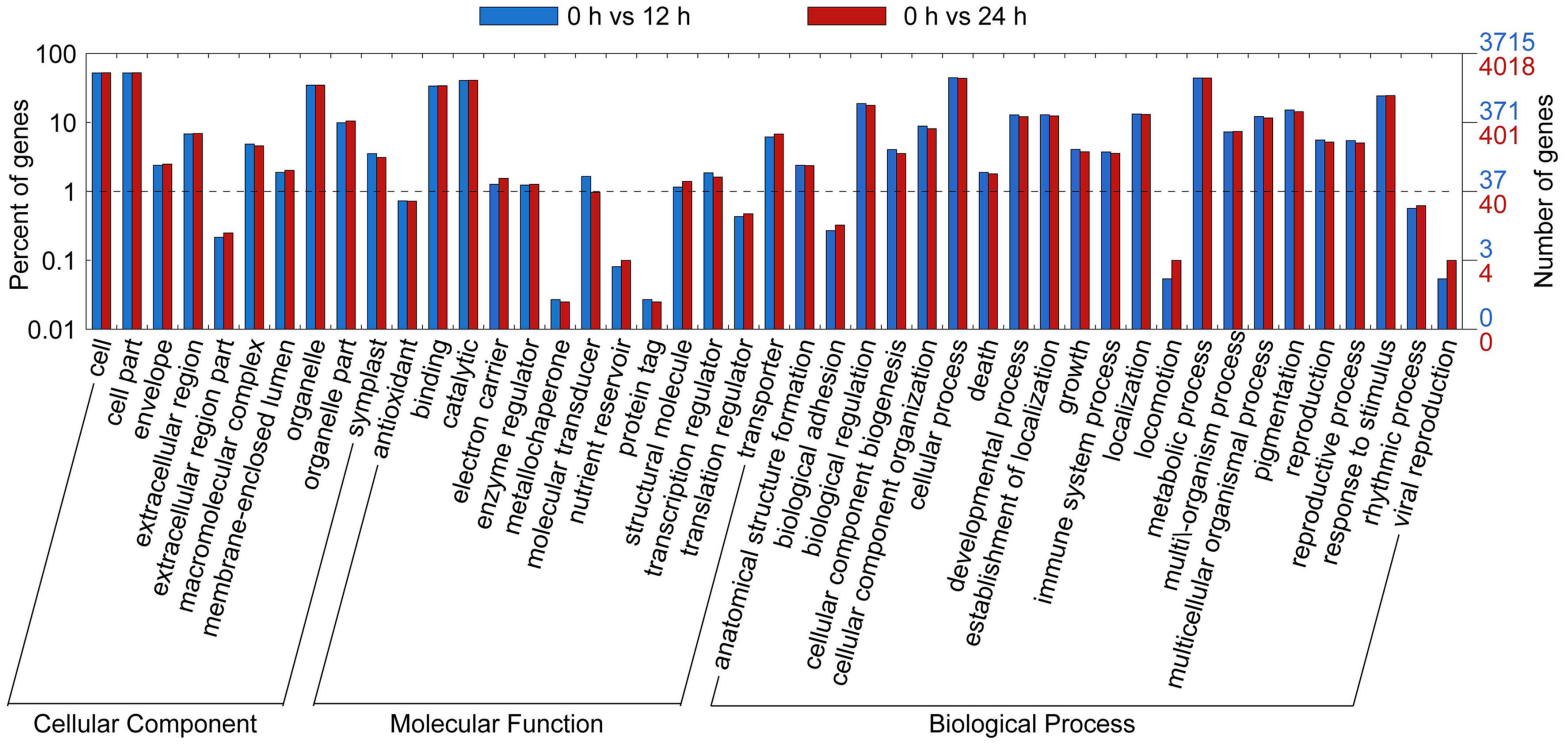
**Gene Ontology (GO) functional classification analysis of DETs based on RNA-Seq data**. GO functional classification analysis of DETs in 0 vs. 12 h and 0 vs. 24 h. Based on sequence homology, 2,573 differentially expressed genes could be categorized into three main categories (biological process, cellular component, and molecular function), which include 25, 13, and 12 functional groups, respectively.

Gene ontology functional classification analyses were performed to classify the functions of the DETs during SA treatment. Based on sequence homology, all DETs could be categorized into 38 functional groups at time points 12 and 24 h (Figure 3). In the biological process (BP) category, the largest groups were metabolic processes, cellular process, and single-organism process. In the cellular component (CC) category, the greatest numbers of genes were found in the cell part and cell terms. In the molecular function (MF) category, most of the DETs were mapped into the catalytic activity and binding groups.

**Figure 4:**
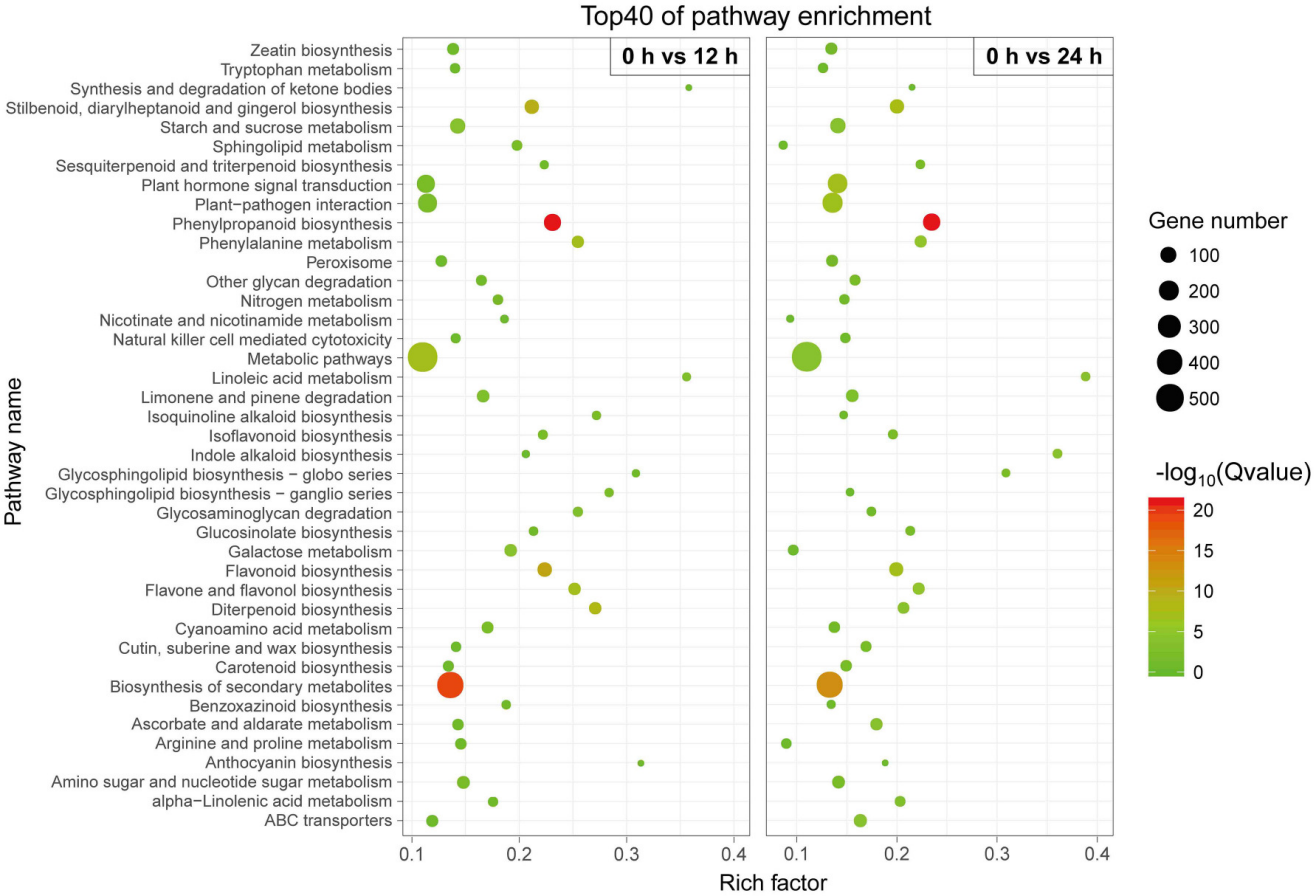
**Top 40 enriched KEGG pathways among the annotated DEGs across two comparisons**. The *Y*-axis on the left represents KEGG pathways, and the *X*-axis indicates the enrichment factor. Low *q*-values are shown in green, and high *q*-values are depicted in red.

We conducted a KEGG-pathway-based analysis to obtain a better understanding of the biological functions of these DETs. Among these DETs with a KEGG pathway annotation, 3,504 DETs were identified in the 0 h-vs.-12 h library and mapped onto 120 KEGG pathways (Table S3), while 3,537 DETs were identified in the 0 h-vs.-24 h library and mapped onto 121 KEGG pathways (Table S4). To determine whether genes involved in secondary metabolites were enriched, the KEGG pathway database was searched using the DETs to reveal the top 40 significantly enriched pathways. The following KEGG pathways were enriched after the 12 h SA treatment: 127 DETs were enriched in “phenylpropanoid biosynthesis,” 439 DETs were related to “biosynthesis of secondary metabolites,” 73 DETs were related to “flavonoid biosynthesis,” and 41 DETs were related to “phenylalanine metabolism” (Figure 4, Table S3). All 14 DETs related to “phenylalanine, tyrosine and tryptophan biosynthesis” were up-regulated after SA treatment (Figure S6), suggest that SA may stimulate accumulation of the two precursors in phenylpropanoid biosynthesis. Furthermore, 18 DETs were found to be associated with the “tyrosine metabolism.” Among these, more than 88% of the DETs were up-regulated (Figure S7). Similar results were observed for the 12 h SA treatment sample (Figure 4, Table S4). These results indicate that phenylalanine and tyrosine biosynthesis pathways were up-regulated by SA treatment, the increased precursors maybe a cause of improving accumulation of acteoside.

### Analysis of acteoside biosynthesis

**Figure 5:**
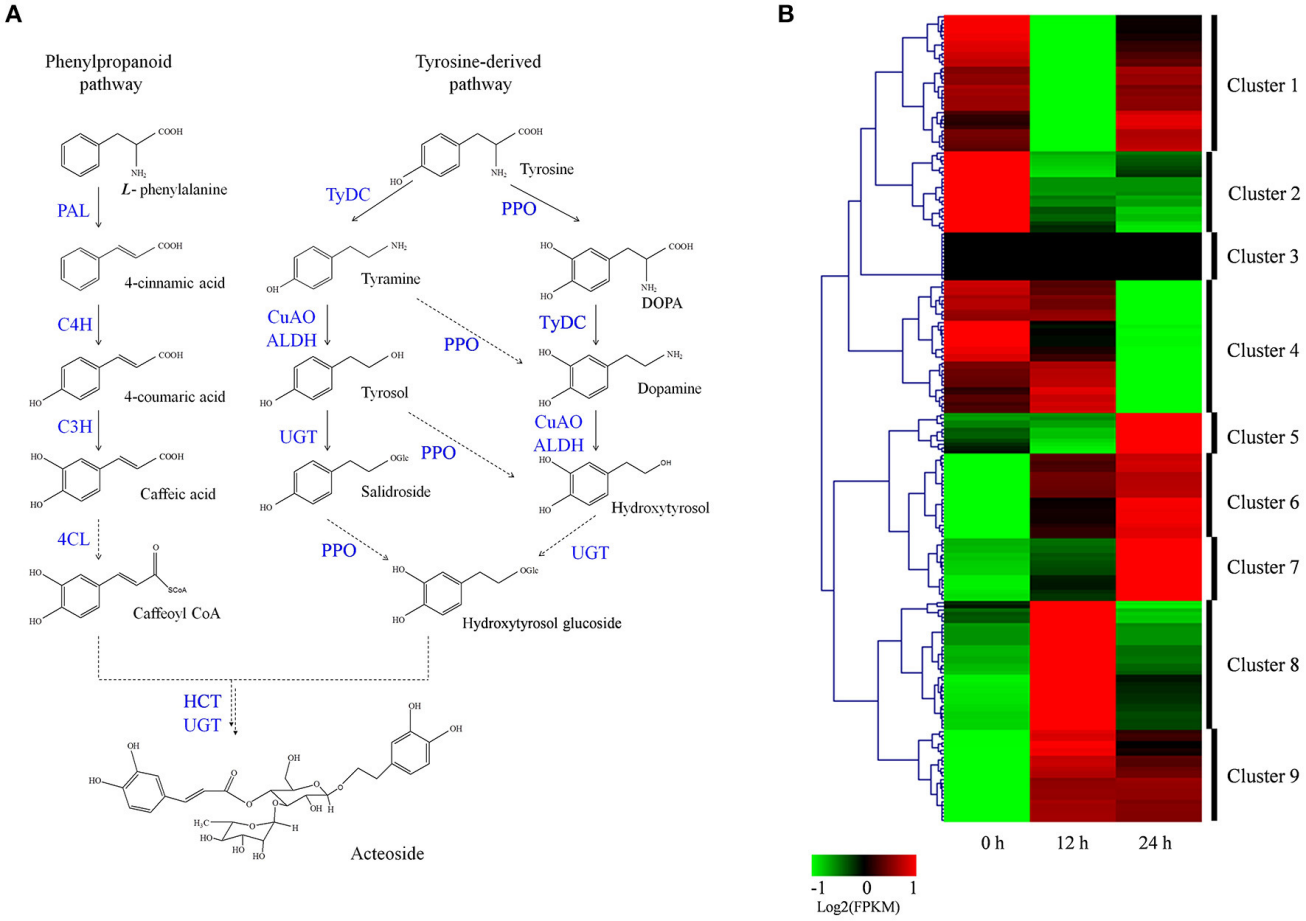
**Putative acteoside biosynthesis pathway and expression level of associated unigenes across in ***R. glutinosa*** hairy roots with SA treated**. **(A)** Proposed pathway for acteoside biosynthesis. **(B)** Expression levels of the identified unigenes were annotated as enzyme coding genes from acteoside biosynthesis pathways. The expression value (FPKM) for unigenes were log_2_ transformed and scaled across each row, and heatmap was generated by MultiExperiment Viewer (MeV). PAL, phenylalanine ammonia-lyase; C4H, cinnamate-4-hydroxylase; C3H, coumarate-3-hydroxylase; TyrDC, tyrosine decarboxylase; PPO, polyphenol oxidase; CuAO, copper-containing amine oxidase; ALDH, alcohol dehydrogenase; UGT, UDP-glucose glucosyltransferase; 4CL, 4-coumarate-CoA ligase; HCT, Shikimate O-hydroxycinnamoyltransferase.

Acteoside biosynthesis begins with the generation of phenylalanine and tyrosine precursors by the shikimate pathway (Alipieva et al., 2014). The hydroxytyrosol moiety of acteoside is synthesized from tyrosine through either tyramine and/or dopamine, whereas its caffeoyl moiety is synthesized from phenylalanine via a cinnamate pathway (Ellis, 1983; Saimaru and Orihara, 2010). The BAHD acyltransferase superfamily encodes proteins catalyzing the acyl-transfer from coenzyme A-activated acids to varying acceptor molecules (D'Auria, 2006). Therefore, we inferred that shikimate O-hydroxycinnamoyltransferase (HCT) maybe involved in acteoside biosynthesis. Using the annotated *R. glutinosa* transcriptome assembly, we identified 219 unigenes with sequence lengths of more than 200 bps, annotated as known enzymes involved in the pathway for acteoside biosynthesis (Figure 5A; Table S5). All of the genes encoding enzymes involved in the biosynthesis of the acteoside were present in our *R. glutinosa* transcriptome (Table 2). In most cases, more than one unigene was annotated as the same enzyme. Such unigenes may represent different fragments of a single transcript, different members of a gene family, or both.

**Table 2:** **The numbers of unigene involved in acteoside biosynthesis of ***R. glutinosa*****.

**Enzyme name**	**Enzyme code**	**Number of unigenes**
Phenylalanine ammonia-lyase (PAL)	EC:4.3.1.24	6
Cinnamate-4-hydroxylase (C4H)	EC:1.14.13.11	5
Coumaroylquinate (coumaroylshikimate) 3′-monooxygenase (C3H)	EC:1.14.13.36	4
4-coumarate-CoA ligase (4CL)	EC:6.2.1.12	14
Tyrosine/DOPA decarboxylase (TyDC)	EC:4.1.1.25	10
Polyphenol oxidase (PPO)	EC:1.14.18.1	14
Copper-containing amine oxidase (CuAO)	EC:1.4.3.21	6
Alcohol dehydrogenase (ALDH)	EC:1.1.1.1	53
UDP-glucose glucosyltransferase (UGT)	EC:2.4.1.-	58
Shikimate O-hydroxycinnamoyltransferase (HCT)	EC:2.3.1.133	49
Total		219

To identify which of these genes are important for acteoside biosynthesis, we performed hierarchical clustering analysis. The 219 unigenes were placed into nine clusters based on their expression patterns in hairy roots treated with SA (Figure 5B). In cluster 6 and 9, gene expression significantly increased and reached the highest expression level at the 12 or 24 h time point after SA treatment (Figure 5B; Figure S8), indicating that the expression of cluster 6 and 9 genes coincides with acteoside accumulation. In clusters 5, 7, and 8, the expression level of unigenes was up-regulated only at the 12 or 24 h time points. In contrast, the genes in clusters 1, 2, and 4 were down-regulated at at least one time point. Oddly, the FPKM-values of genes in cluster 3 were always zero at the three time points, indicating these genes did not expressed in hairy roots. We noted that similar expression trends exhibited for some genes under both 12 and 24 h SA treatment were identical to the above-mentioned enzyme families commonly expressed in both genotypes, but the transcript expressed was different.

To identify genes displaying significant expression changes during SA treatment, DETs were analyzed by comparing 12 and 24 h libraries with the control library. A total of 54 unigenes were found to be differentially expressed at at least one of the time points after SA treatment (Table S6). Surprisingly, all DETs were significantly up-regulated genes, and none were significantly down-regulated. Forty-one and thirty DETs exhibited a significant increase in expression levels at 12 and 24 h time points, respectively. There were 17 up-regulated DETs in both the 12/0 h and 24/0 h comparisons. Among the 54 identified acteoside biosynthesis genes, 14 of them encode 5 enzymes predominantly expressed in the early stage (12 h) of SA treatment. They include five phenylalanine ammonia-lyase (*PAL*) genes, three copper-containing amine oxidase (*CuAO*) genes, and two each of the cinnamate-4-hydroxylase (*C4H*), coumaroylquinate (coumaroylshikimate) 3′-monooxygenase (*C3H*) and tyrosine decarboxylase (*TyDC*) genes. In contrast, most of the *ALDH* genes (8/10) had higher expression levels at the late stage (24 h) after SA treatment. Sixteen DETs encoding four enzymes involved in acteoside biosynthesis were significantly up-regulated at both 12 and 24 h time points. They include four *4CL* genes, three polyphenol oxidase (*PPO*) genes, five UDP-glucosyl transferase (*UGT*) genes, and four Shikimate *HCT* genes.

### Transcript profiles of candidate genes involved in acteoside biosynthesis

**Figure 6:**
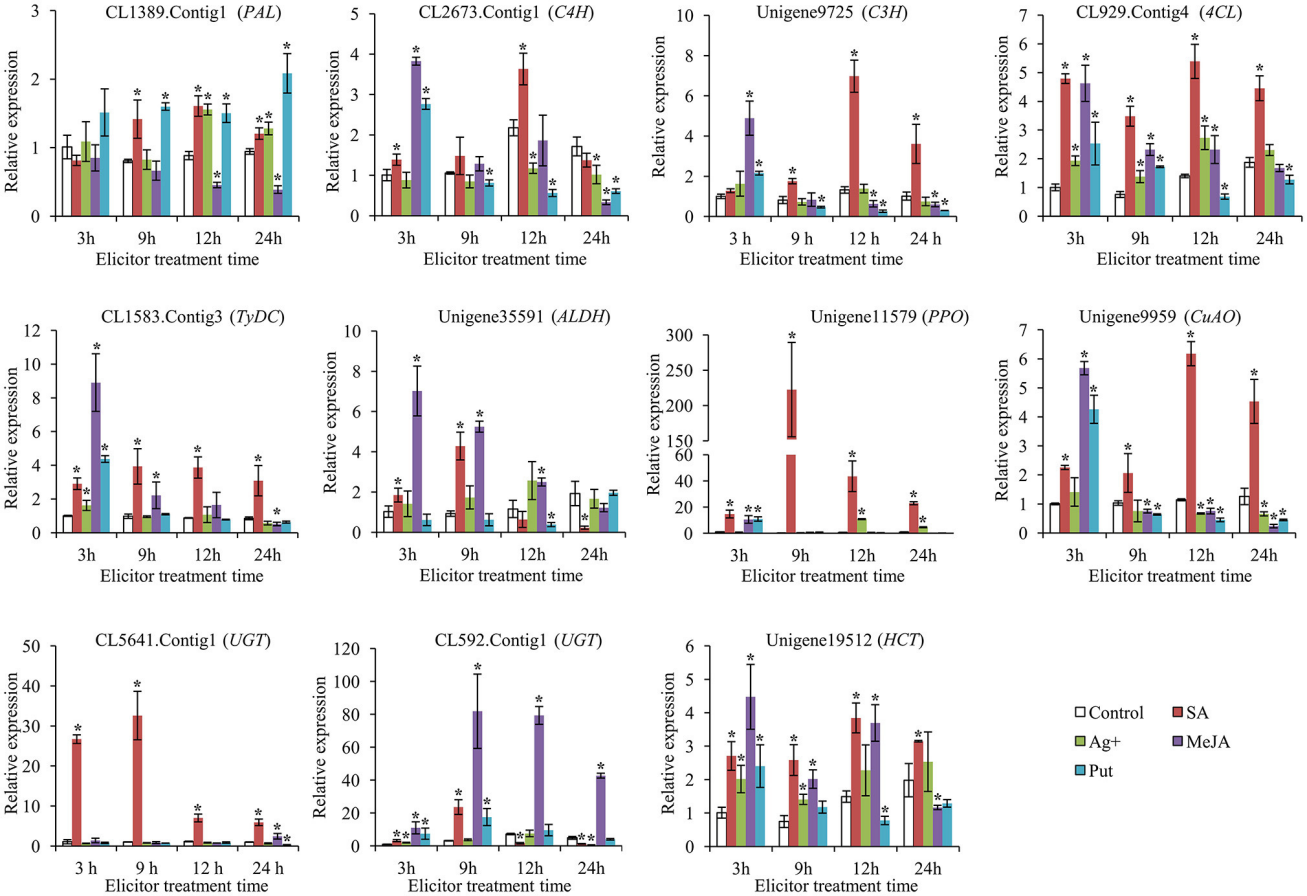
**Expression of key enzyme genes involved in biosynthesis of acteoside after SA, Ag^**+**^, MeJA, and Put treatments**. These genes expression level were all determined by Real-time PCR. Vertical bars indicate the standard deviation of three biological replicates. Asterisks indicate a significant difference at the *p* < 0.05 level.

To gain a more comprehensive understanding of the modes of action of SA, MeJA, Ag^+^, and Put in affecting the accumulation of acteoside in *R. glutinosa* hairy roots, the expression levels of genes encoding enzymes belonging to the biosynthesis pathway of acteoside were examined by qRT-PCR during the 24 h period after elicitation. *RgTIP41* gene was stably expressed in roots, stems, leaves and flower tissues of *R. glutinosa*, and has been used as internal reference gene for research the expression characteristics of *R. glutinosa* coding genes (Sun et al., 2014; Wang et al., 2015). Gene expression levels were normalized using *RgTIP41* as the reference gene as internal standard, and the transcripts of all associated genes in the control were set to 1. The results of gene transcript analysis are shown in Figure 6, indicating that all the selected genes investigated were up-regulated by SA treatment, although the time and degree of response differed. Under SA treatment, the expression levels of CL1389.Contig1 (*PAL*), CL2673.Contig1 (*C4H*) and Unigene9725 (*C3H*) were observed to gradually increase and reached peak values at 12 h with transcript levels 1.6-, 3.6-, and 7.0-fold higher, respectively, than those of the control. The relative up-regulated levels of CL1583.Contig3 (*TyDC*), Unigene35591 (*ALDH*), Unigene11579 (*PPO*), CL5641.Contig1 (*UGT*), and CL592.Contig1 (*UGT*) after 9 h SA treatment were the most significant, reaching 3.9-, 4.3-, 222.6-, 32.5-, and 23.6-fold, respectively. CL929.Contig4 (*4CL*), Unigene9959 (*CuAO*) and Unigene19512 (*HCT*) showed a similar expression trend: the largest difference between treatments and control occurred at 12 h after treatment, reaching 5.4-, 6.2-, and 3.8-fold, respectively. In addition, Unigene11579 (*PPO*) and CL5641.Contig1 (*UGT*) exhibited significantly higher transcriptional levels than other genes, indicating that they might be more sensitive and critical than other genes for acteoside biosynthesis in hairy roots following SA elicitation.

Under MeJA treatment, most of the investigated genes (10 genes) were up-regulated at at least one time point (Figure 6). The largest relative difference in the expression level of CL2673.Contig1 (*C4H*), Unigene9725 (*C3H*), CL929.Contig4 (*4CL*), CL1583.Contig3 (*TyDC*), Unigene35591 (*ALDH*), Unigene11579 (*PPO*), Unigene9959 (*CuAO*), and Unigene19512 (*HCT*) between treatments and control occurred at 3 h after treatment, reaching 3.8-, 4.9-, 4.6-, 8.9-, 7.0-, 10.6-, 5.7-, and 4.5-fold, respectively. Furthermore, CL592.Contig1 (*UGT*) was up-regulated to ~81.9- and 79.3-fold after being treated for 9 and 12 h, respectively. The expression profiles of these selected genes suggested that they may promote acteoside accumulation in hairy roots of *R. glutinosa* treated with MeJA. However, after Ag^+^ and Put treatments, the expression levels of the major selected genes showed only slight up-regulation at some time points or were even down-regulated, indicating that Ag^+^ and Put did not induce the transcription of these genes to increase acteoside accumulation in hairy root of *R. glutinosa*.

**Figure 7:**
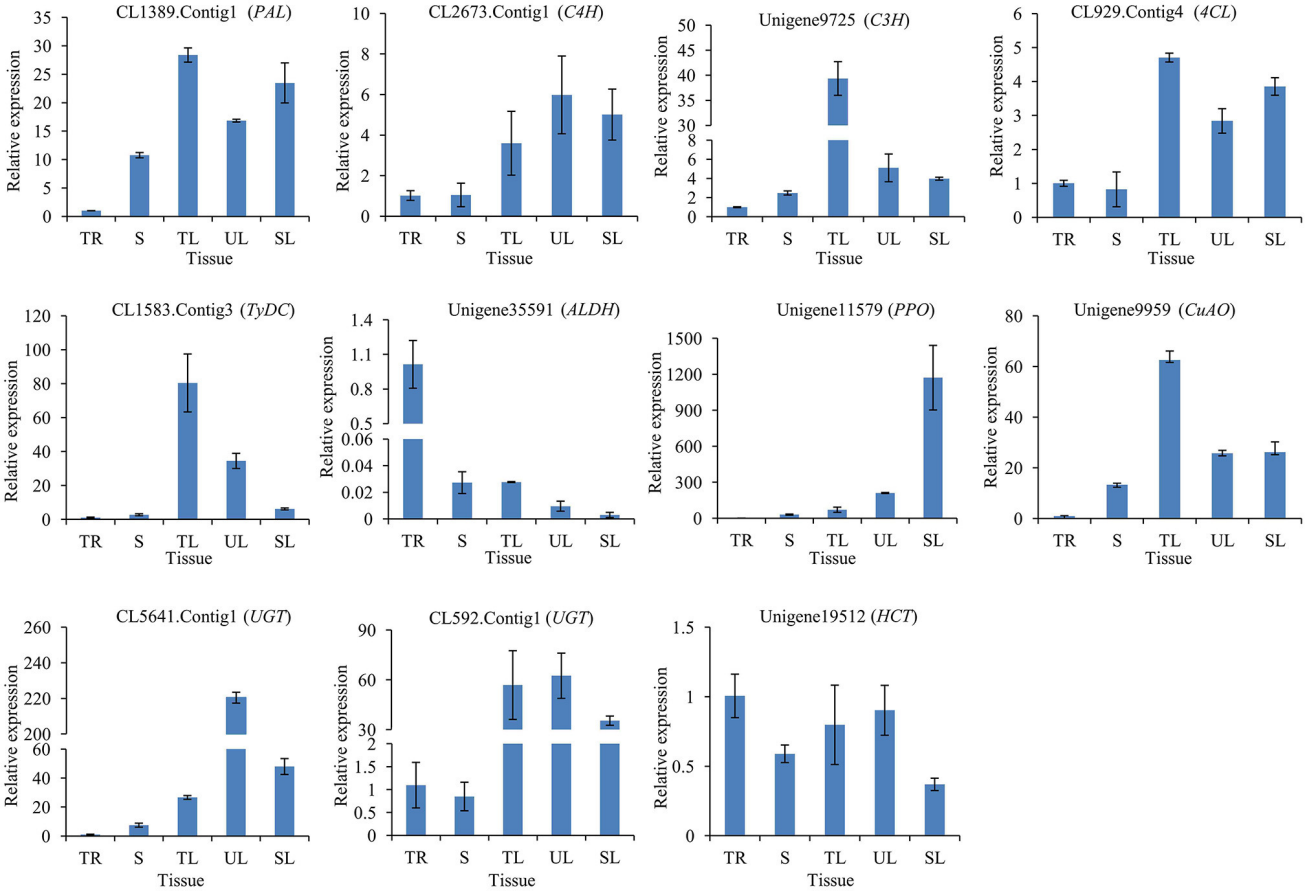
**Expression of candidate genes involved in biosynthesis of acteoside across five tissues of ***R. glutinosa*****. These genes expression level were all determined by Real-time PCR. Vertical bars indicate the standard deviation of three biological replicates. TR, Tuberous root; S, Stem; TL, Tender leaf; UL, Unfolded leaf; SL, Senescing leaf.

To illuminate the relative expression of enzyme-encoding genes with acteoside accumulation, we detected relative expression levels of the 11 candidate genes in the tuberous root (TR), stem (S), tender leaf (TL), unfolded leaf (UL), and senescing leaf (SL) of *R. glutinosa* by qRT-PCR. This analysis revealed that CL1389.Contig1 (*PAL*), CL2673.Contig1 (*C4H*), Unigene9725 (*C3H*), CL929.Contig4 (*4CL*), CL1583.Contig3 (*TyDC*), Unigene11579 (*PPO*), Unigene9959 (*CuAO*), CL5641.Contig1 (*UGT*), CL592.Contig1 (*UGT*), and Unigene19512 (*HCT*) showed higher expression levels in the leaf (including TL, UL, and SL) than in the tuberous root (Figure 7), consistent with the acteoside content in the tuberous root, which was significantly lower than that of the leaf for *R. glutinosa* cultivars Wen 85-5 and QH (Figure S9A).

**Figure 8:**
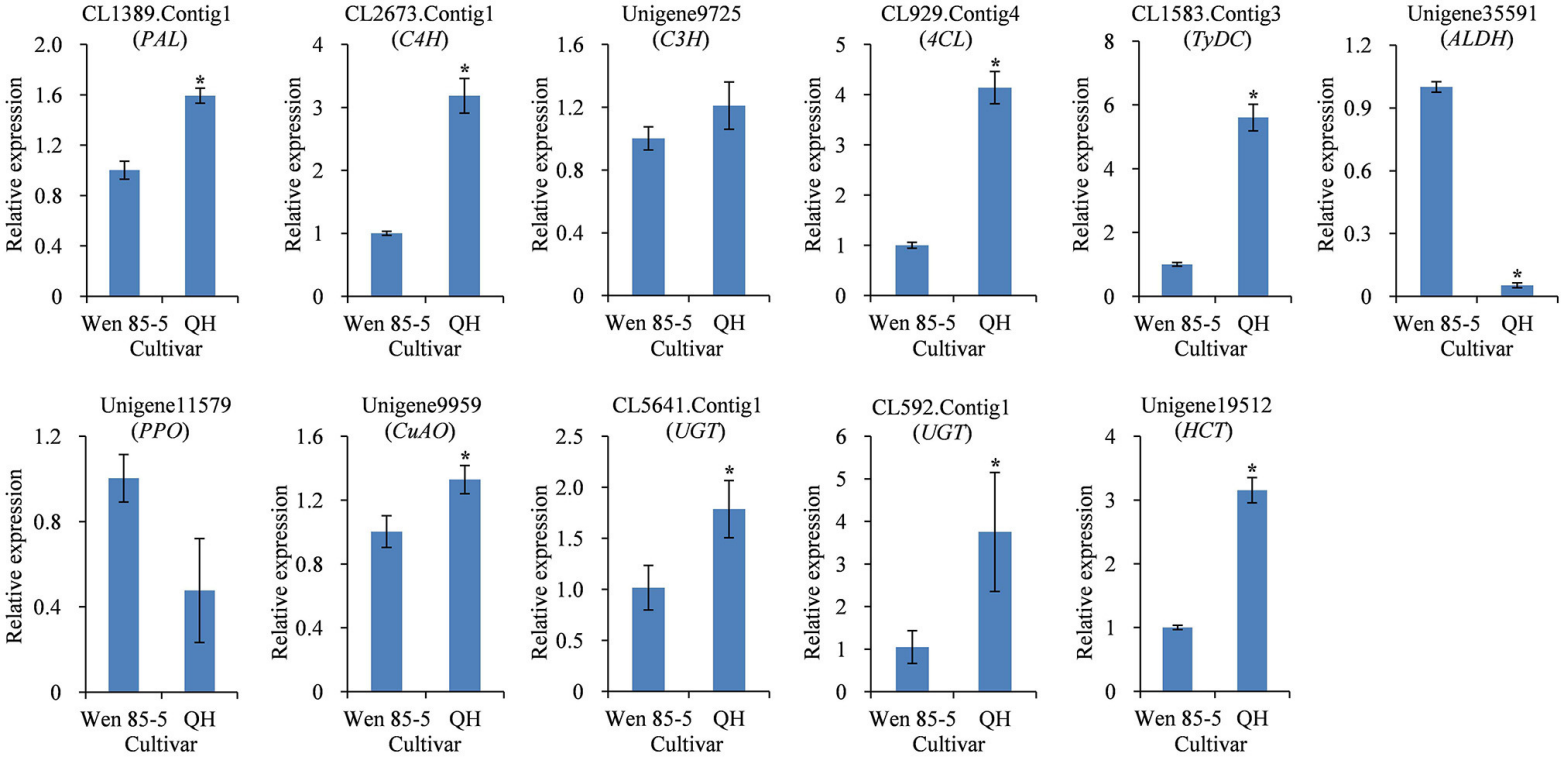
**Expression of candidate genes involved in biosynthesis of acteoside in tuberous roots of Wen 85-5 and QH at 30 July, 2015**. These genes expression level were all determined by Real-time PCR. Vertical bars indicate the standard deviation of three biological replicates. Asterisks indicate a significant difference at the *p* < 0.05 level.

The secondary metabolite content in many medicinal plants has been found to vary significantly with genotype (Alagna et al., 2012; Song and Li, 2015), reflecting genetic control by the plant. QH is a high-acteoside (HA) *R. glutinosa* cultivar with a higher content of acteoside (up to 1.61 mg/g dw) in tuberous roots at three harvest times (Figure S9B). In contrast, Wen 85-5 is a low-acteoside (LA) cultivar with a lower content of acteoside (lower than 0.69 mg/g) in tuberous roots (Figure S9B). To detect the expression levels of the 11 candidate genes in Wen 85-5 and QH tuberous roots, qRT-PCR was performed. The results showed that most of candidate genes, including CL1389.Contig1 (*PAL*), CL2673.Contig1 (*C4H*), CL929.Contig4 (*4CL*), CL1583.Contig3 (*TyDC*), Unigene9959 (*CuAO*), CL5641.Contig1 (*UGT*), CL592.Contig1 (*UGT*), and Unigene19512 (*HCT*) had higher expression levels in QH than in Wen 85-5 (Figure 8). The identification of candidate genes highly expressed in high acteoside cultivars may provide an insight into the relationship between genotype and acteoside biosynthesis.

## Discussion

The overall pathway of acteoside biosynthesis was first researched in *O. europaea* cells by isotope-labeled precursor feeding experiments, which identified the phenylpropanoid and tyrosine-derived pathways as being involved (Saimaru and Orihara, 2010). In the phenylpropanoid pathway, phenylalanine is transformed into caffeic acid by PAL, C4H and C3H. In the tyrosine-derived pathway, tyrosine is metabolized to hydroxytyrosol through PPO (also referred as tyrosinase), TyDC, CuAO, and ALDH, or through TyDC, CuAO, ALDH, and UGT (Saimaru and Orihara, 2010; Alagna et al., 2012). The classical tyrosinases belong to a group of copper proteins, PPOs, which exhibit both monophenol monooxygenase activity (EC: 1.14.18.1) and o-diphenol oxidase activity (EC: 1.10.3.1; Steiner et al., 1999). In the tyrosine-derived pathway, PPO can catalyze tyrosine to DOPA, tyramine to dopamine, tyrosol to hydroxytyrosol, or salidroside to hydroxytyrosol glucoside, although which of these is the main pathway has yet to be established. In pathway of acteoside biosynthesis, several intermediates leading from caffeic acid, salidroside, hydroxytyrosol, glucose, and rhamnose to acteoside still need to be screened, and key enzymes and their encoding genes need to be further studied.

The acylation of oxygen- and nitrogen-containing substrates to produce esters and amides, respectively, is one of the most common types of modification of secondary metabolites, in which a large family of BAHD acyltransferases play important roles (D'Auria, 2006). For example, *Rauvolfia serpentine* vinorine synthase plays a key role in the production of the ajmalan type of monoterpene indole alkaloids (Bayer et al., 2004). Rosmarinic acid synthase (RAS) is a member of the BAHD acyltransferases, which couple 4-coumaroyl-CoA and 3,4-dihydroxyphenyllactic acid to form 4-coumaroyl-3′4′-dihydroxyphenyllactic acid (4C-DHPL) (Berger et al., 2006; Ma et al., 2015). The molecular phylogenetic tree, based on a multiple sequence alignment of 7 *R. glutinosa* HCTs and 27 UGTs from other plants, showed that Unigene19512, *Salvia miltiorrhiza* RAS, *Coleus blumei* RAS and *Arabidopsis thaliana* HCT belong to group I (Figure S10). The glycosyltransferases (GTs) may lead to glycoside formation and transfer nucleotide-diphosphate-activated sugars to low-molecular-weight substrates. The activated sugar form is usually UDP-glucose but can also be UDP-galactose or UDP-rhamnose (Vogt and Jones, 2000). Six *R. glutinosa* UGT genes were up-regulated with SA treatment, only four of them possessed complete opening reading frames (ORF). Phylogenetic analysis of the deduced protein sequences with UGTs from other plants revealed that CL5641.Contig1 belong to the UGT73 group, Unigene12663 to the UGT90A1 group, Unigene15683 to the UGT79B group, and CL592.Contig1 to the UGT83A1 group (Figure S11). Many members of UGT73 group involved in plant secondary metabolism, such as UGT73C6 (Jones et al., 2003), *CaUGT2* (Kaminaga et al., 2004), UGT73C5 (Poppenberger et al., 2005), and UGT73A17 (Ohgami et al., 2014). Therefore, we infer that caffeic acid is transformed into caffeoyl CoA by 4CL, that hydroxytyrosol is transformed into hydroxytyrosol glucoside through UGT, or that salidroside is transformed into hydroxytyrosol glucoside through PPO; then, caffeoyl CoA and hydroxytyrosol glucoside are further coupled by the HCT to form acteoside (Figure 5A).

Plant secondary metabolism, derived from their primary metabolism, has arisen from interactions between plants and their growth environments (including biotic and abiotic environmental conditions) during a long period of evolution. However, the yield of plant secondary metabolites in plant cells is low, which has been a major obstacle for the production of plant secondary metabolites. Through several decades of efforts, various elicitor signal compounds and abiotic stresses have been successfully applied to improve the yield of such plant secondary metabolites (Zhao et al., 2005). For example, arachidonic acid (AA), SA, MeJA, ammonium citrate (AC), and AgNO_3_ can increase the yield of Taxol, although their mechanisms of action have been shown to be different (Yuan et al., 2002). Ethephon enhances both root growth and ginsenoside accumulation in *Panax ginseng* at 50 μmol/L but inhibits ginsenoside accumulation at 100 μmol/L (Bae et al., 2006). Yeast (YE) was reported to strongly improve the accumulation of rosmarinic acid (RA) and phenolics in *S. miltiorrhiza* hairy roots at 200 and 400 mg/L, but Ag^+^ was only detectable at 15 mmol/L (Yan et al., 2006). In a new report, SA was found to increase the yield of RA in cell cultures of *S. miltiorrhiza* (Guo et al., 2015).

To date, studies on stimulating the accumulation of PeGs by adding elicitors have focused primarily on the medicinal plant *Cistanche deserticola* (Lu and Mei, 2003; Xu et al., 2005; Cheng et al., 2006; Chen et al., 2007). In previous reports, SA, MeJA, Ag^+^, and Put all increased the content of PeGs in *C. deserticola* cell cultures at suitable elicitor concentrations. In this report, we found that SA and MeJA could increase accumulation of acteoside in *R. glutinosa* hairy roots, but SA was much more effective than MeJA in stimulating the accumulation of acteoside (Figure 1B). SA is a well-known inducer of plant systematic acquired resistance in plant-pathogen (Jian et al., 2005), and often used to improve the accumulation of plant secondary metabolites (Hao et al., 2014; Saini et al., 2014). The content of total PeG of *C. deserticola* cell cultures was obviously elevated by SA at concentration range of 10 ~ 100 μmol/L (Xu et al., 2005). In this study, *R. glutinosa* hairy roots treated with 25 μmol/L SA attained an acteoside content of 11.66 mg/g dw after SA treatment, which was 2.28-fold higher than the control (Figure 1B). Exogenous application of MeJA to the plant cell culture or intact plant stimulates biosynthesis of secondary metabolites (Jian et al., 2005). The stimulating effect of MeJA on ginsenosides accumulation in suspension culture of *Panax ginseng* roots was stronger at a larger dose (200 μmol/L) (Ali et al., 2006). However, MeJA increased the PeG content in *C. deserticola* cell cultures only at very lower dose (1~10 μmol/L; Xu et al., 2005). Similar to the effect of MeJA on *C. deserticola* cell cultures, the lower dose (5 μmol/L) of MeJA increased the acteoside content of *R. glutinosa* hairy root, and higher dose (25 μmol/L) inhibited its accumulation (Figure 1B). In consideration of the negative effect of DMSO on secondary metabolite accumulation, we will further study the effect of MeJA on acteoside content in *R. glutinosa* hairy roots with DMSO added control. Put had no effect on acteoside accumulation, whereas Ag^+^ decreased the acteoside content (Figure 1B). This result may suggest that the acteoside accumulation in *R. glutinosa* occurs through different regulation mechanisms than in other plant species.

Gene-to-metabolite networks in *Catharanthus roseus* cells revealed that the gene expression was involved in metabolite accumulation (Rischer et al., 2006). Recently, transcriptome approaches have become powerful tools to screen candidate genes involved in the biosynthesis of secondary metabolites in plants, particularly in non-model species. Much research has aimed to illuminate the complex biosynthesis pathways of functional ingredients in medicinal plants through the use of RNA-seq digital gene expression analysis, and many secondary metabolism genes have been identified from plants with little genomic sequence information, including *Siraitia grosvenorii* (Tang et al., 2011), *Momordica cochinchinensis* (Hyun et al., 2012), *Uncaria rhynchophylla* (Guo et al., 2014), *Pyrus communis* (Yang et al., 2015), *Eleutherococcus senticosus* (Hwang et al., 2015), and *S. miltiorrhiza* (Gao et al., 2014; Xu et al., 2015, 2016).

Elicitors may change enzymatic activity, regulate the expression of related genes, and then enhance the accumulation of plant secondary metabolites. MeJA and Ag^+^ could improve mRNA levels of kaurene synthase-like (KSL) and copalyl diphosphate synthases (CPS) in *S. miltiorrhiza* hairy roots, which in accordance with the biosynthesis of tanshinone IIA (Gao et al., 2009). MeJA could stimulate ginsenoside accumulation in adventitious root cultures of *Panax quinquefolium*, transcriptomic analysis indicated that four P450 genes (c15743_g1, c52011_g1, c39772_g1, c55422_g1), and one UDP-xylose synthases (c52571_g3) showed a significant up-regulation in response to MeJA, which were likely to be involved in ginsenoside biosynthesis (Wang et al., 2016). In the present study, we identified 219 unigenes annotated as known enzymes involved in the acteoside biosynthesis pathway of *R. glutinosa* (Table 2). Transcriptome analysis revealed 54 unigenes differentially expressed at least one of the time points after SA treatment, and 16 differentially expressed unigenes were significantly up-regulated at both the 12 and 24 h time points (Table S6), which may interpret the relationship of candidate genes expression to acteoside accumulation. To further investigate the expression characteristics of the selected candidate genes in acteoside biosynthesis pathway, their expression abundances in response to SA, MeJA, Ag^+^, and Put treatments were detected by qRT-PCR method. The results indicated that the transcription levels of all tested genes in acteoside biosynthesis pathway were improved by SA treatment, but the intensity of the increase was different (Figure 6). The highest increase in response to abiotic elicitors was observed for Unigene11579 (*PPO*), followed by the two *UGTs* (CL5641.Contig1 and CL592.Contig1). The results suggest that SA induced acteoside accumulation in *R. glutinosa* hairy roots by activating the expression of key enzyme encoding genes involved in acteoside biosynthesis. Furthermore, most of the investigated genes (10 genes) were up-regulated at at least one time point of MeJA treatment (Figure 6), which provided molecular evidence for MeJA elevated acteoside content in *R. glutinosa* hairy roots. However, the effects of Ag^+^ and Put on expression levels of selected genes were weaker (Figure 6), which well-accord with the acteoside accumulation (Figure 1).

Biosynthesis and accumulation of secondary metabolite are often tissue-specific, and related genes of enzymes and regulators also show organ- or tissue-specific expression patterns (Upadhyay et al., 2014; Garg et al., 2015). For example, the accumulation of tanshinones in the rhizome (Wang and Wu, 2010), qRT-PCR analysis demonstrated that *SmCPS* and *SmKSL* were higher expressed in the rhizome than above ground tissues of *S. miltiorrhiza* (Guo et al., 2013). Using co-regulation analysis, CYP76AH1 was identified as a key enzyme in tanshinones biosynthesis (Guo et al., 2013). In this research, acteoside content and gene expression levels were detected in tuberous roots and leaves of *R. glutinosa* Wen 85-5 and QH. It showed that acteoside concentrations in leaves were often ten times as high as in tuberous roots and sometimes more (Figure S9A). Most of investigated candidate genes (91%, 10/11) showed higher expression levels in the leaves than in the tuberous roots (Figure 7). The results provide molecular insight into tissue-specific accumulation of acteoside in *R. glutinosa*.

There was significant genotypical difference on content of active ingredients in medicinal plants (Yuan et al., 2012b; Yang et al., 2016). Using the high content and low content cultivars as materials for further transcriptional analyses might be effective methods for identification of candidate genes involved in compounds metabolism. The mRNA levels of 35 olive transcripts involved in the pathways of the main secondary metabolites were detected on fruits of high- and low-phenolic *O. europaea* varieties, the result showed that a strong correlation was observed between phenolic compound concentrations and transcripts putatively involved in their biosynthesis (Alagna et al., 2012). Transcriptome analysis of tubers from elite purple-flesh cultivar and conventional white-flesh cultivar of yam were performed, as a result, a number of candidate genes which are possibly involved in purple-flesh tuber formation were revealed (Wu et al., 2015). qRT-PCR results indicated that the eight selected candidate unigenes had higher expression levels in the HA cultivar QH than in the LA cultivar Wen 85-5 (Figure 8). The positive correlations between acteoside content and gene expression suggest a critical role for these candidate unigene encoding enzymes related to acteoside biosynthesis.

## Conclusion

In this research, we established a hairy root culture system for *R.-glutinosa*-screened SA as an optimal elicitor that can dramatically stimulate acteoside accumulation in hairy roots. RNA-seq analysis was performed on SA-treated hairy roots. GO and pathway enrichment analyses revealed that these DETs are involved in the acteoside biosynthesis pathway and that gene expression alterations in this pathway might be responsible for the increased content of acteoside in *R. glutinosa*.

## Author contributions

FW, JZ, and ZZ conceived and designed the research. FW, JZ, YS, LW, and CX performed the experiments. FW, ML, and HS generated the pictures. FW, JZ, and ZZ wrote and revised the manuscript. BZ, JD, and LG contribute reagents/materials and interpretation of the results. All authors have read and approved the final draft.

### Conflict of interest statement

The authors declare that the research was conducted in the absence of any commercial or financial relationships that could be construed as a potential conflict of interest.

## Supplementary material

Figure S1 **PCR amplification of ***rolB*** and ***rolC*** genes in ***Ri*** plasmid transformed ***R. glutinosa*** plants cv. Wen 85-5**. Lanes 1–5, transformed hairy root lines; M, DNA Marker; P, Plasmid DNA (positive control); NT, non-transformed wild type root (negative control).

Figure S2 **Biomass of ***R. glutinosa*** hairy roots in liquid MS medium. (A)** Fresh weight. **(B)** Dry weight.

Figure S3 **Effect of SA on acteoside production in ***R. glutinosa*** hairy roots at 12 and 24 h elicitation**. The vertical bars show the *SD*-values (*n* = 3). The asterisks indicate statistically significant differences at *p* < 0.05.

Figure S4 **Venn diagram analysis of the quantity of the up-regulated (A)** and down-regulated **(B)** DETs identified at different time points of SA treatment.

Figure S5 **Correlation analysis between DET and qRT-PCR data**.

Figure S6 **Differentially expressed genes involved in phenylalanine, tyrosine, and tryptophan biosynthesis signaling pathway under SA treatment**. Genes up-regulated after SA treated from 0 to 12 h are marked in red.

Figure S7 **Differentially expressed genes involved in the tyrosine metabolism signaling pathway under SA treatment**.

Figure S8 **Expression profiles for unigenes from acteoside biosynthesis pathways after SA treatment**. The expression profile of 23 and 25 genes in cluster 6 and 9 of Figure 2B, respectively. The expression value (FPKM) for unigenes were log_2_ transformed and scaled across each row, and heatmap was generated by MeV 4.9.0.

Figure S9 **Contents of acteoside in leaves and tuberous roots of ***R. glutinosa***. (A)**, Acteoside contents in leaves and tuberous roots from Wen 85-5 and QH. **(B)**, Acteoside contents in tuberous roots of Wen 85-5 at different harvest times.

Figure S10 **Phylogenetic analysis of HCTs from ***R. glutinosa*** and other plants**. Eg, *Erythranthe guttata*; Nt, *Nicotiana tabacum*; Si, *Sesamum indicum*; Vv, *Vitis vinifera*; At, *Arabidopsis thaliana*; Ms, *Medicago sativa*; Sm, *Salvia miltiorrhiza*; Cb, *Coleus blumei*.

Figure S11 **Phylogenetic analysis of UGTs from ***R. glutinosa*** and other plants**. The full-length sequences of the UGT proteins were aligned using Clustal Omega, and the phylogenetic tree was constructed using the neighbor-joining method in the MEGA6 software (The same as below). Rs, *Rhodiola sachalinensis*; Ca, *Catharanthus roseus*; At, *Arabidopsis thaliana*; Nt, *Nicotiana tabacum*; Si, *Sesamum indicum*; Vv, *Vitis vinifera*; Eg, *Erythranthe guttata*; Bv, *Barbarea vulgaris*.

Table S1 **List of primers used for qRT-PCR analysis in this study**.

Table S2 **List of up- and down-regulated differentially expressed transcripts during 0 h-vs.-12 h, 0 h-vs.-24 h, and 12 h-vs. -24 h libraries**.

Table S3 **Enriched signaling pathways of DETs detected between 0 and 12 h hairy roots**.

Table S4 **Enriched signaling pathways of DETs detected between 0 and 24 h hairy roots**.

Table S5 **List of transcripts were identified from ***R. glutinosa*** EST database may be involved in acteosdie biosynthesis**.

Table S6 **List of candidate genes involved in acteoside biosynthesis of ***R. glutinosa*****.
