# Supplementary figures and images for: Transcriptome Analysis of Salicylic Acid Treatment in Rehmannia glutinosa Hairy Roots Using RNA-seq Technique for Identification of Genes Involved in Acteoside Biosynthesis
Source: Front Plant Sci. 2017 May 17;8:787. doi: 10.3389/fpls.2017.00787 (PMC5434160; doi:10.3389/fpls.2017.00787)

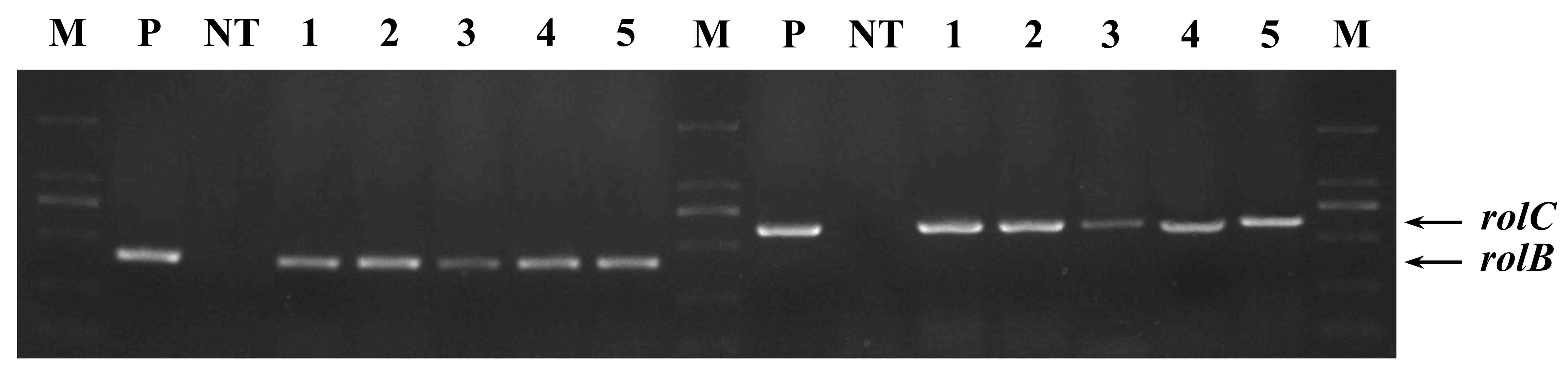

Supplement: Figure S1 — PCR amplification of rolB and rolC genes in Ri plasmid transformed R. glutinosa plants cv. Wen 85-5. Lanes 1–5, transformed hairy root lines; M, DNA Marker; P, Plasmid DNA (positive control); NT, non-transformed wild type root (negative control). [file Image1.JPEG]

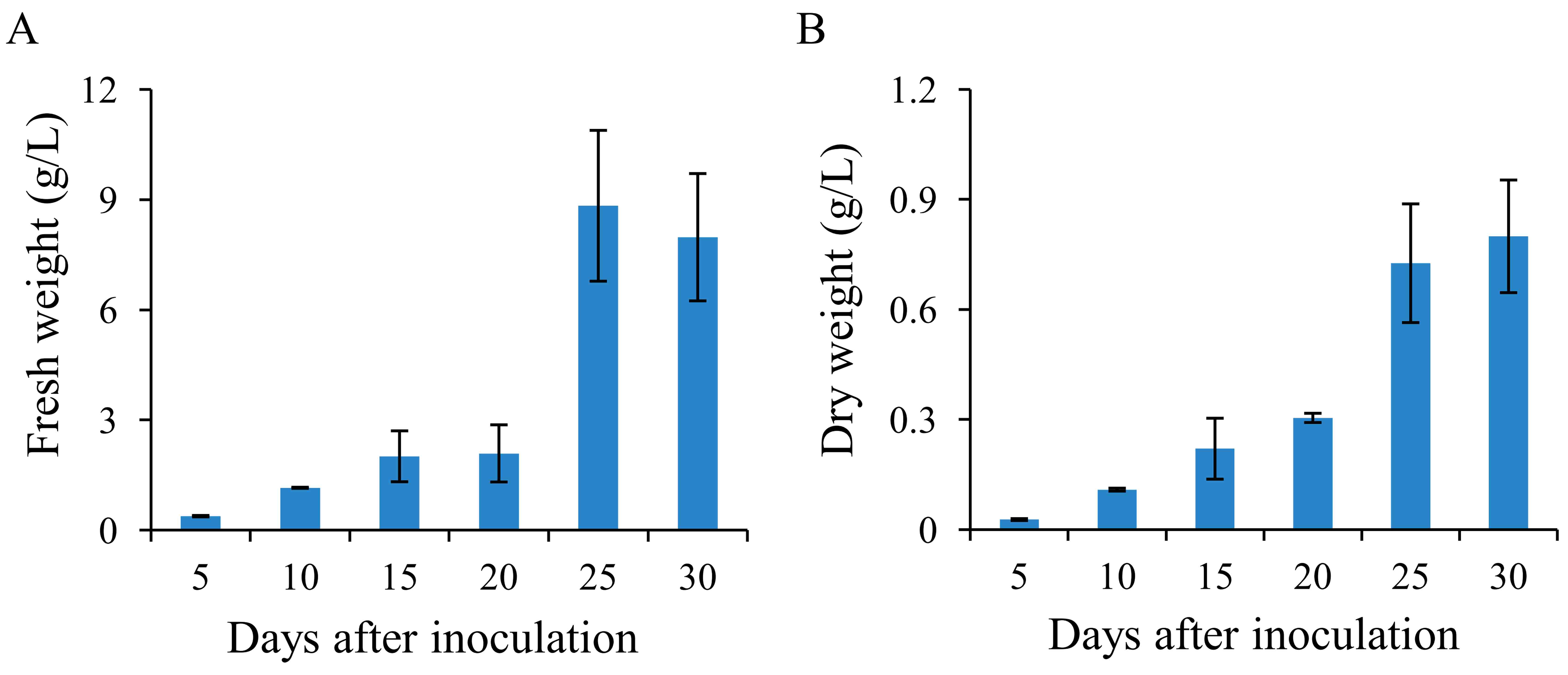

Supplement: Figure S2 — Biomass of R. glutinosa hairy roots in liquid MS medium. (A) Fresh weight. (B) Dry weight. [file Image2.JPEG]

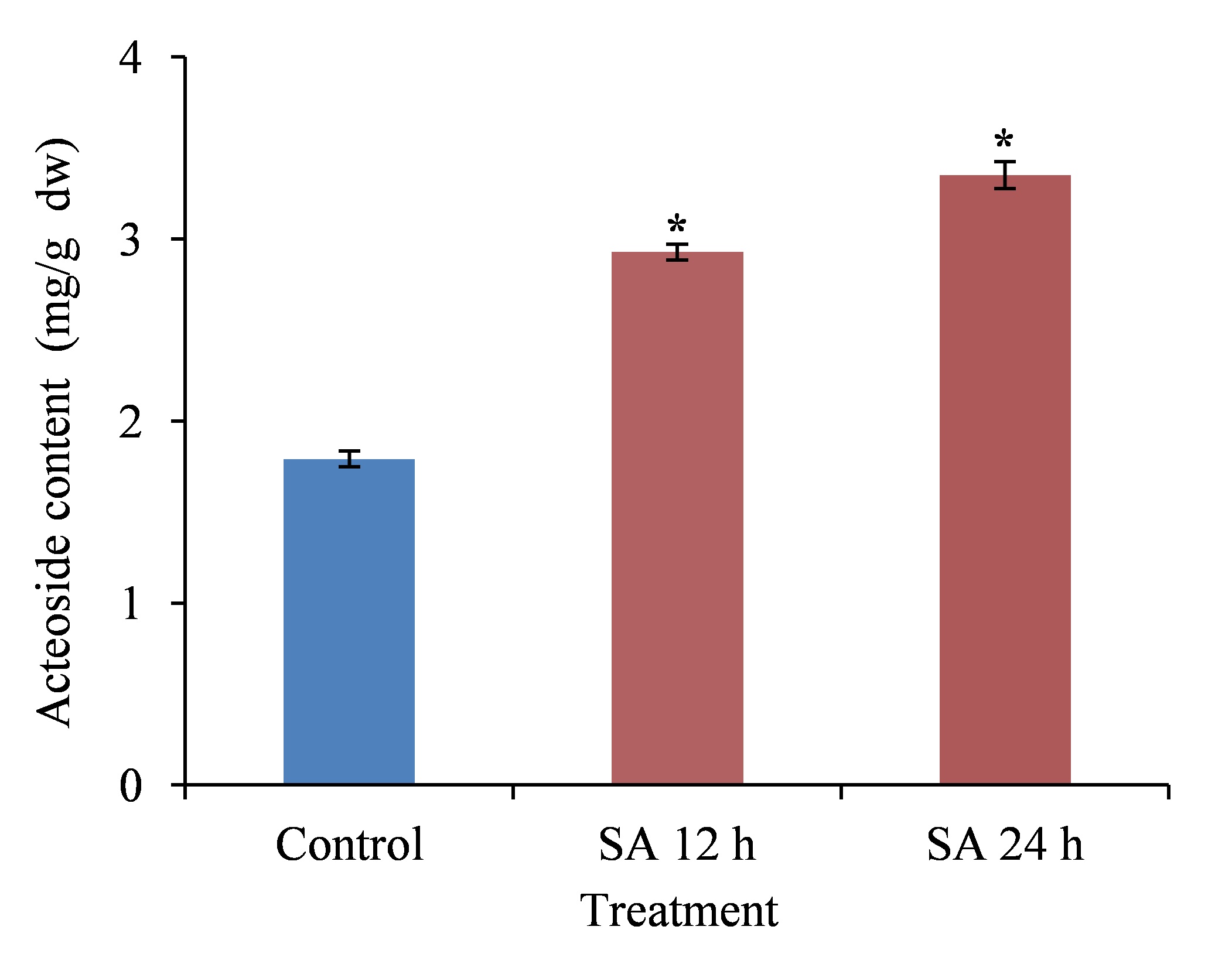

Supplement: Figure S3 — Effect of SA on acteoside production in R. glutinosa hairy roots at 12 and 24 h elicitation. The vertical bars show the SD-values (n = 3). The asterisks indicate statistically significant differences at p < 0.05. [file Image3.JPEG]

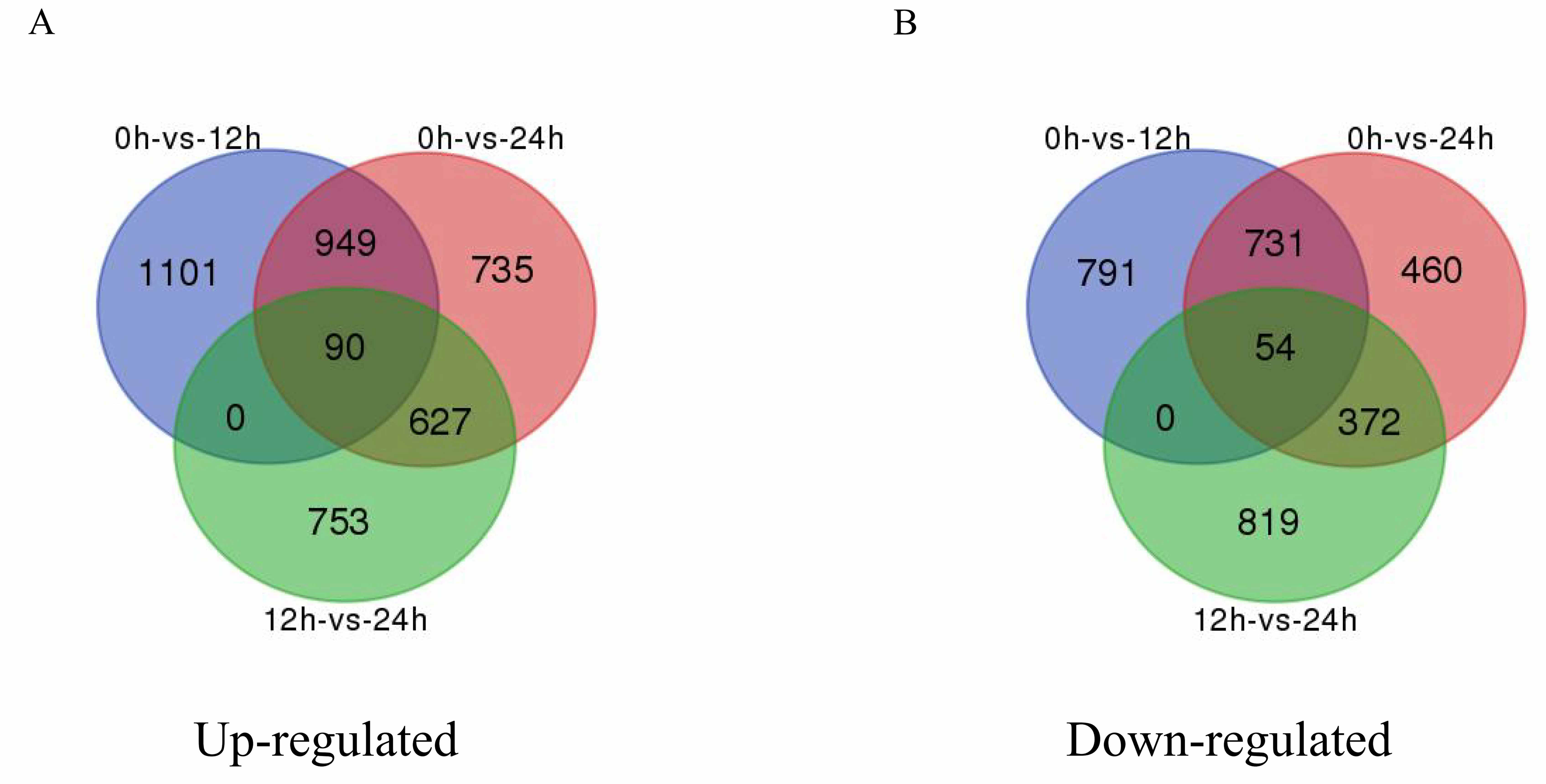

Supplement: Figure S4 — Venn diagram analysis of the quantity of the up-regulated (A) and down-regulated (B) DETs identified at different time points of SA treatment. [file Image4.JPEG]

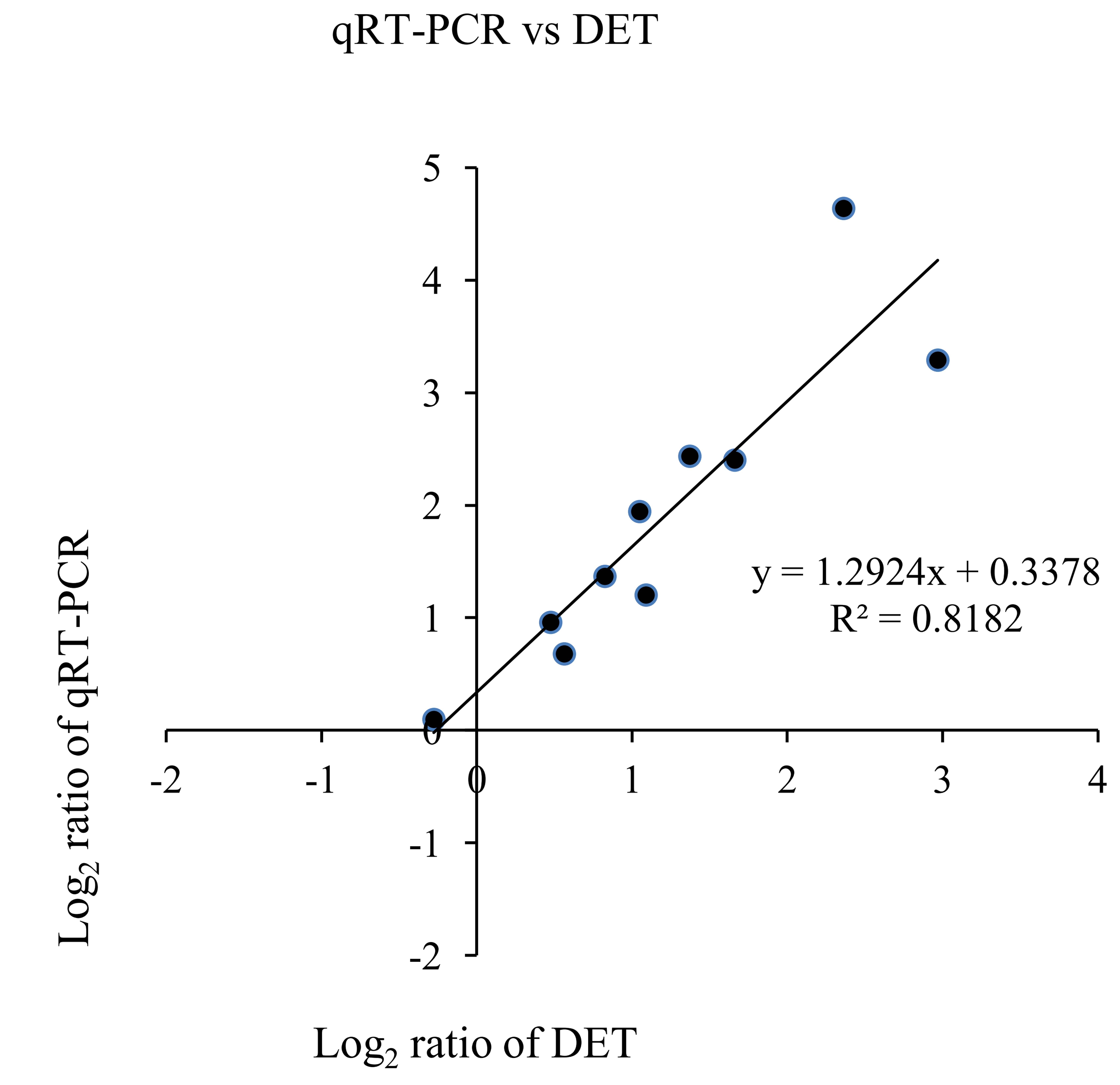

Supplement: Figure S5 — Correlation analysis between DET and qRT-PCR data. [file Image5.JPEG]

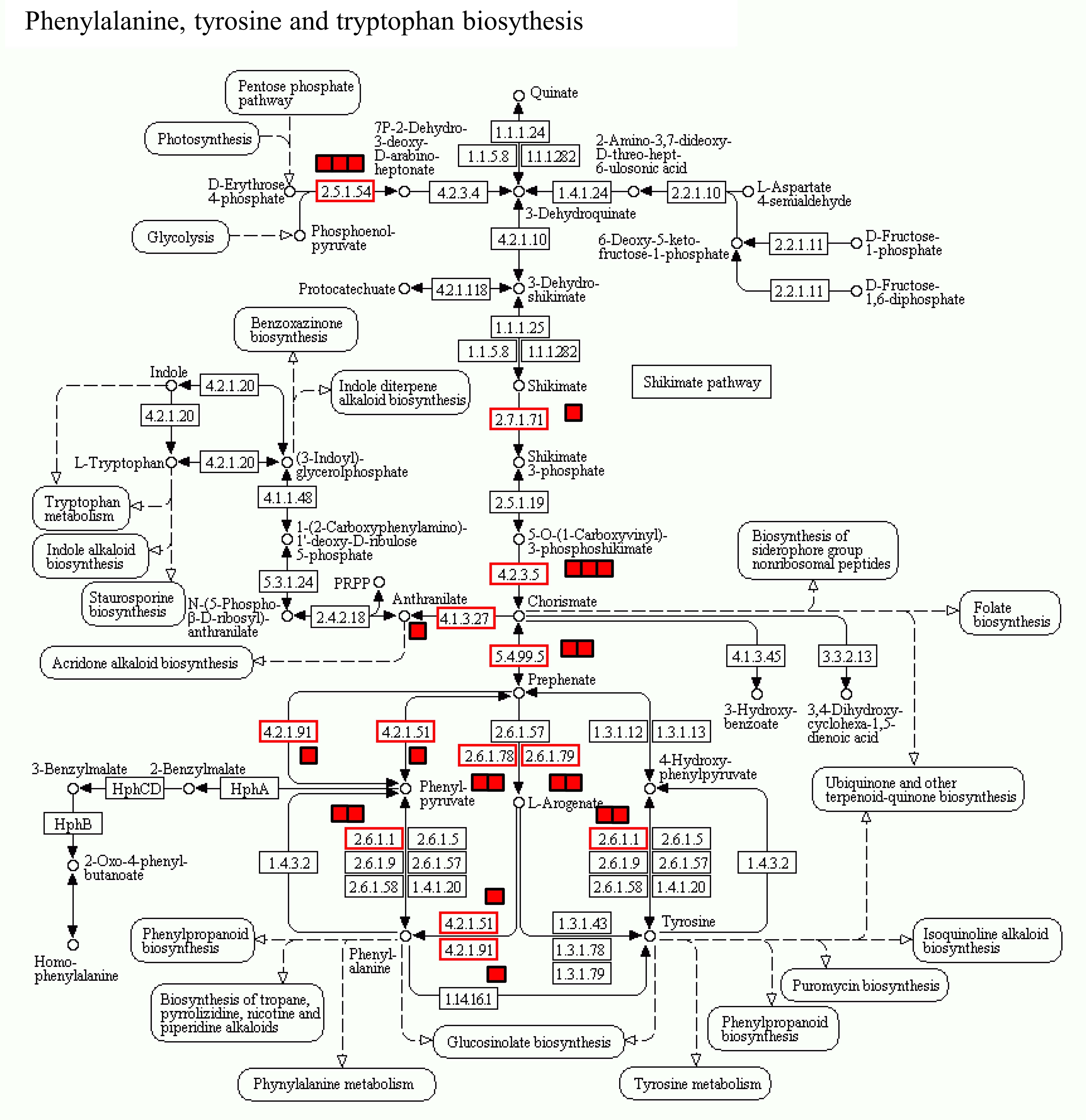

Supplement: Figure S6 — Differentially expressed genes involved in phenylalanine, tyrosine, and tryptophan biosynthesis signaling pathway under SA treatment. Genes up-regulated after SA treated from 0 to 12 h are marked in red. [file Image6.JPEG]

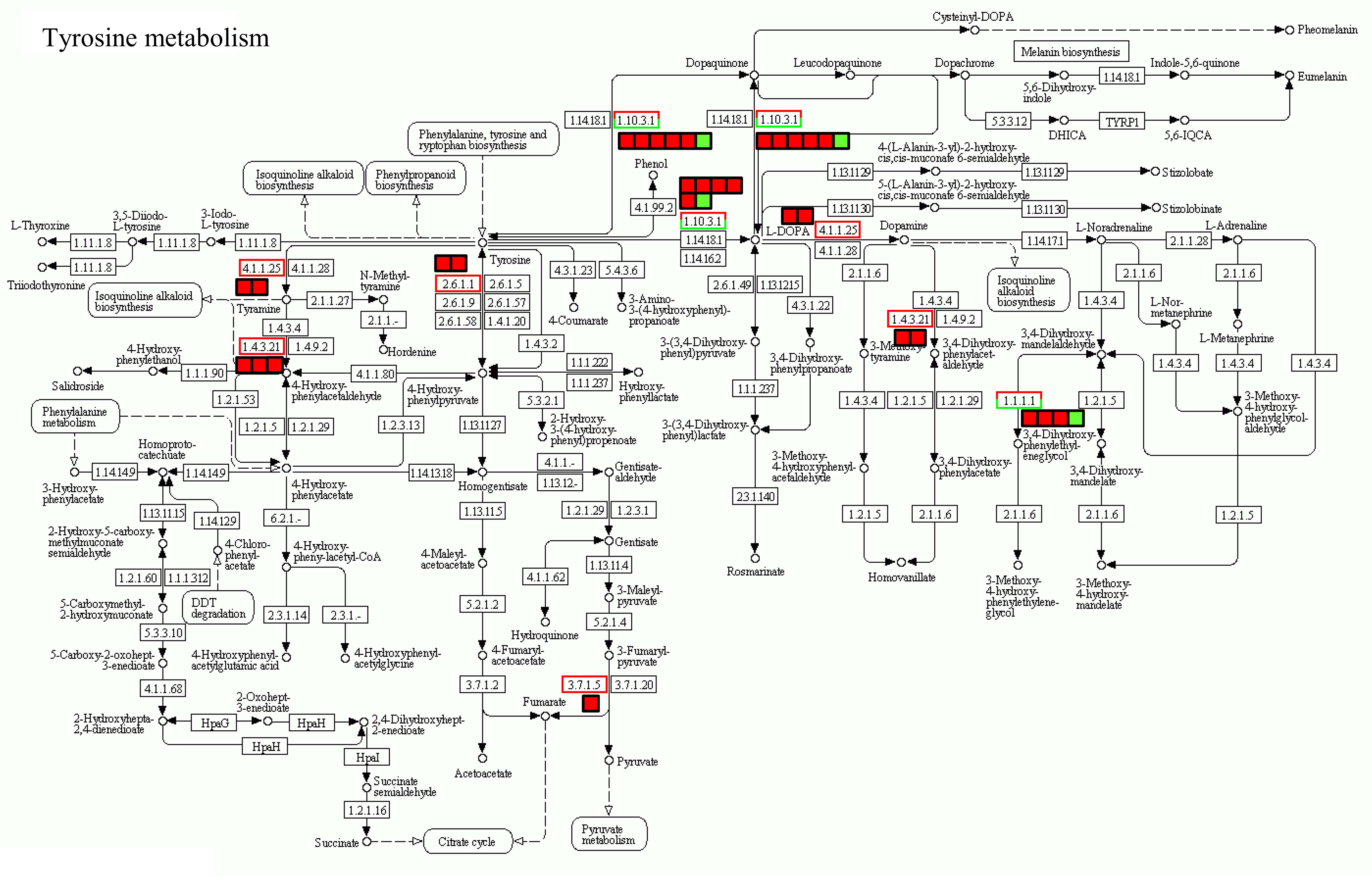

Supplement: Figure S7 — Differentially expressed genes involved in the tyrosine metabolism signaling pathway under SA treatment. [file Image7.JPEG]

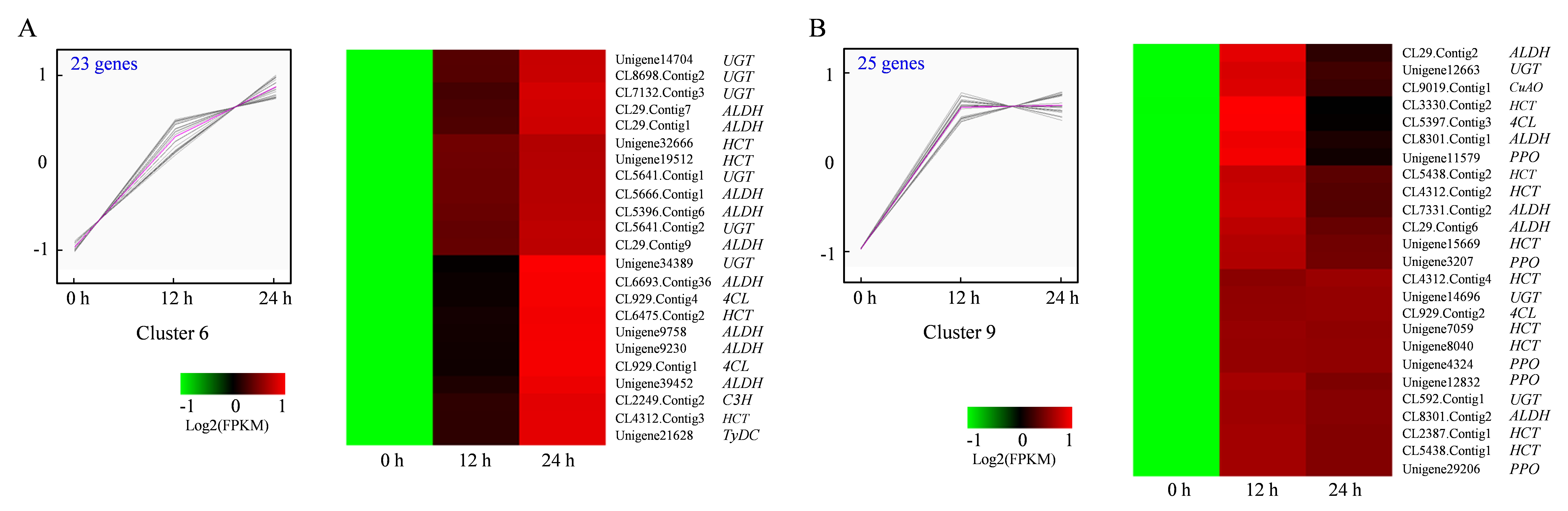

Supplement: Figure S8 — Expression profiles for unigenes from acteoside biosynthesis pathways after SA treatment. The expression profile of 23 and 25 genes in cluster 6 and 9 of Figure 2B, respectively. The expression value (FPKM) for unigenes were log2 transformed and scaled across each row, and heatmap was generated by MeV 4.9.0. [file Image8.JPEG]

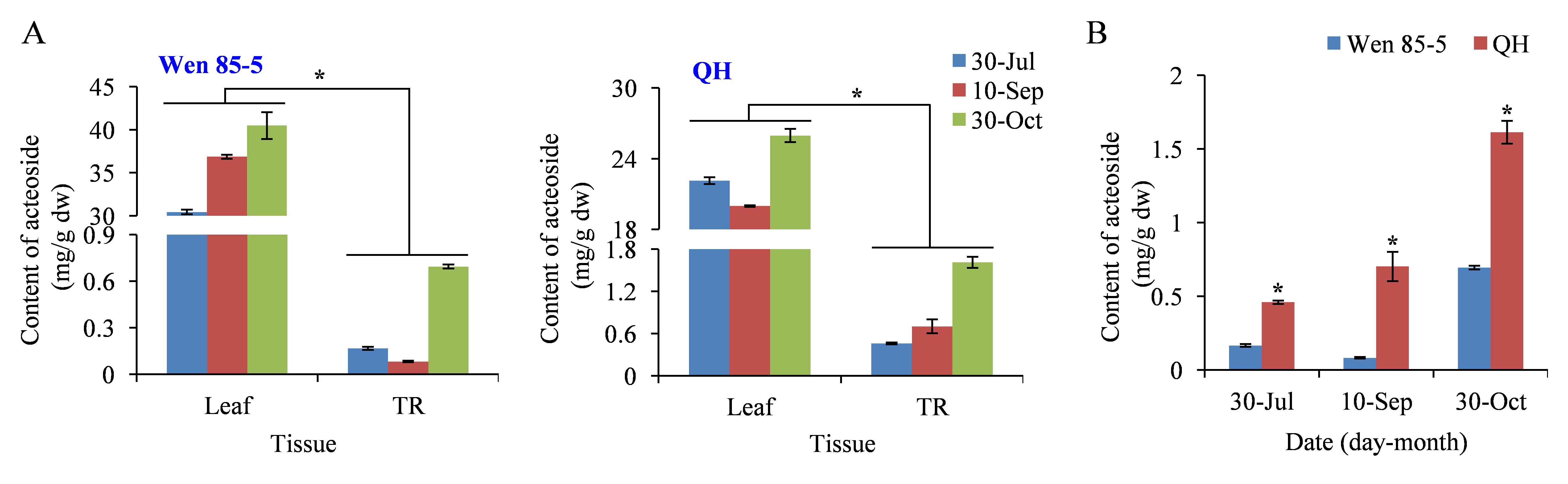

Supplement: Figure S9 — Contents of acteoside in leaves and tuberous roots of R. glutinosa. (A), Acteoside contents in leaves and tuberous roots from Wen 85-5 and QH. (B), Acteoside contents in tuberous roots of Wen 85-5 at different harvest times. [file Image9.JPEG]

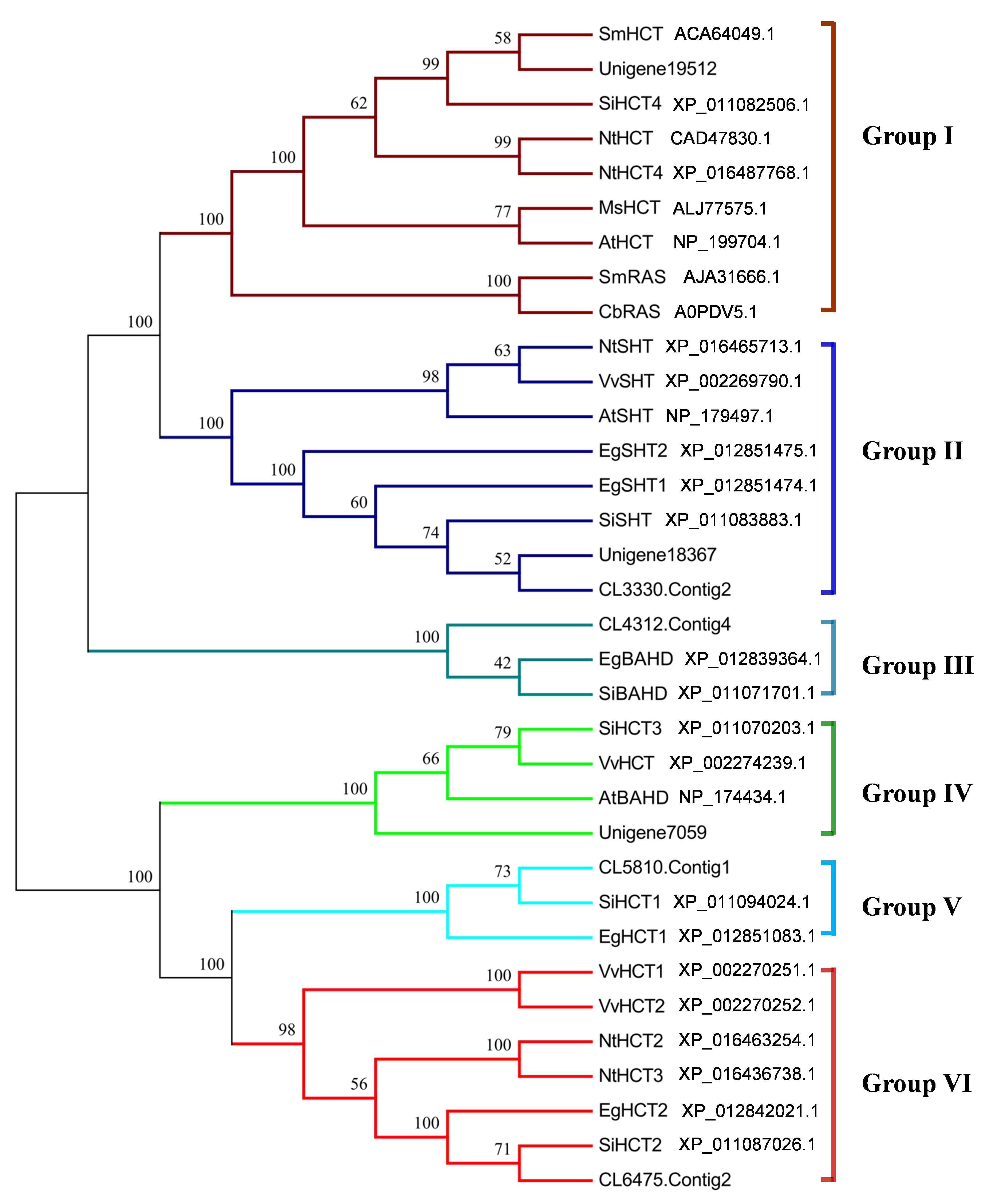

Supplement: Figure S10 — Phylogenetic analysis of HCTs from R. glutinosa and other plants. Eg, Erythranthe guttata; Nt, Nicotiana tabacum; Si, Sesamum indicum; Vv, Vitis vinifera; At, Arabidopsis thaliana; Ms, Medicago sativa; Sm, Salvia miltiorrhiza; Cb, Coleus blumei. [file Image10.JPEG]

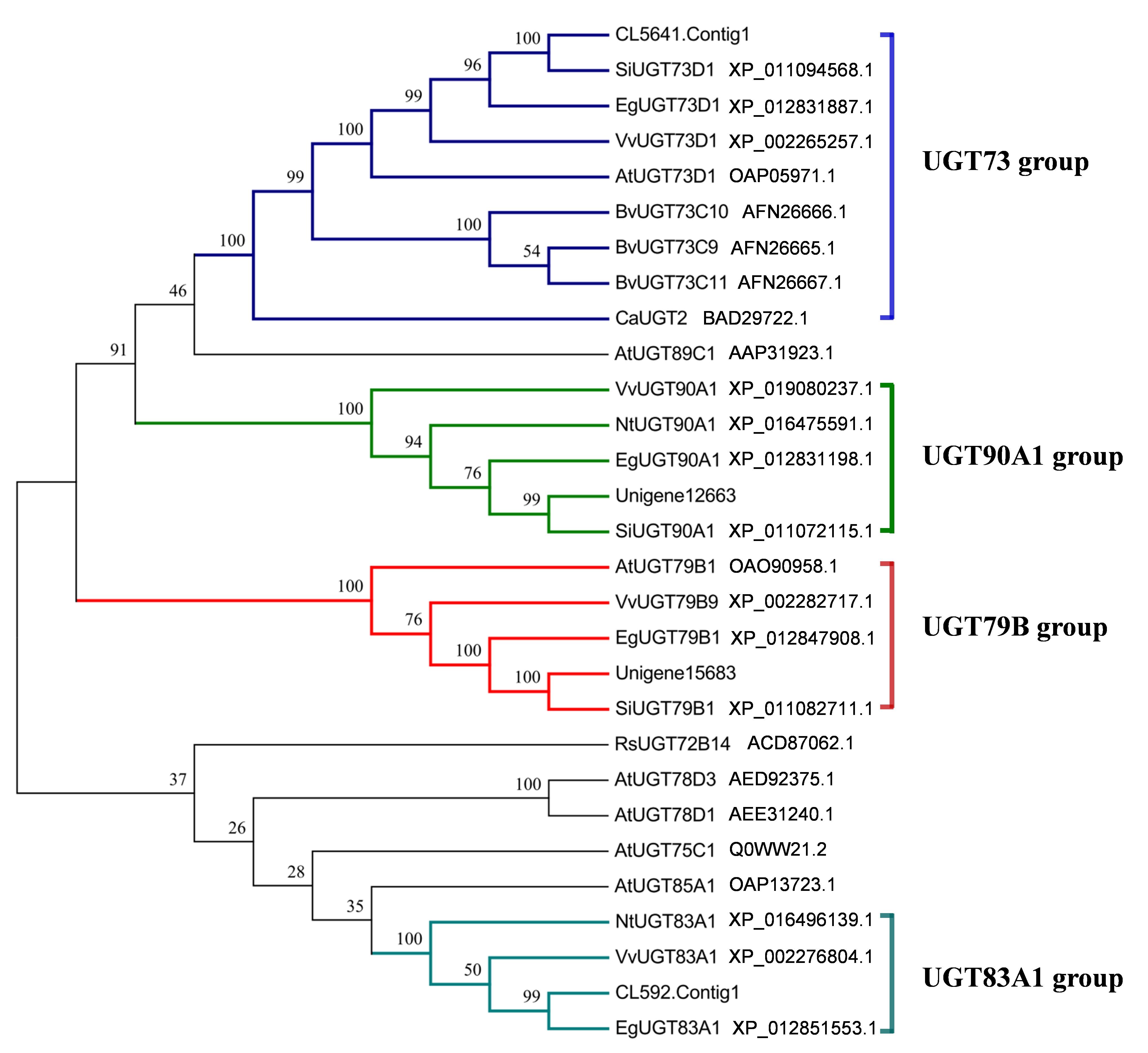

Supplement: Figure S11 — Phylogenetic analysis of UGTs from R. glutinosa and other plants. The full-length sequences of the UGT proteins were aligned using Clustal Omega, and the phylogenetic tree was constructed using the neighbor-joining method in the MEGA6 software (The same as below). Rs, Rhodiola sachalinensis; Ca, Catharanthus roseus; At, Arabidopsis thaliana; Nt, Nicotiana tabacum; Si, Sesamum indicum; Vv, Vitis vinifera; Eg, Erythranthe guttata; Bv, Barbarea vulgaris. [file Image11.JPEG]
